# Machine‐Learning‐Assisted Accurate Prediction of Molecular Optical Properties upon Aggregation

**DOI:** 10.1002/advs.202101074

**Published:** 2021-11-25

**Authors:** Shidang Xu, Xiaoli Liu, Pengfei Cai, Jiali Li, Xiaonan Wang, Bin Liu

**Affiliations:** ^1^ Department of Chemical and Biomolecular Engineering National University of Singapore 4 Engineering Drive 4 Singapore 117585 Singapore; ^2^ Joint School of National University of Singapore and Tianjin University International Campus of Tianjin University Binhai New City, Fuzhou 350207 China

**Keywords:** aggregation‐induced emission, machine learning, molecular design, optical properties, solid‐state materials

## Abstract

For practical applications, molecules often exist in an aggregate state. Therefore, it is of great value if one can predict the performance of molecules when forming aggregates, for example, aggregation‐induced emission (AIE) or aggregation‐caused quenching (ACQ). Herein, a database containing AIE/ACQ molecules reported in the literature is first established. Through training, these machine learning (ML) models can build up the structure–property relationship and thus implement fast prediction of AIE/ACQ properties. To this end, a multi‐modal approach is proposed, multiple prediction methods are compared and designed, and thus an ensemble strategy is developed. First, multiple molecular descriptors are considered at the same time, major features are extracted by dimensionality reduction, and multi‐modal features are synthesized. Then, several state‐of‐the‐art methods are designed and compared to analyze the advantages of the different methods. Finally, the ensemble strategy combines the advantages of the multiple methods to obtain the final prediction result. The reliability of this approach in an unknown molecular space is further verified by three newly designed molecules. Reasonable consistency between model predictions and experimental outcomes is obtained. The result indicates that ML can be a powerful tool to predict molecular properties in the aggregated state, thus accelerating the development of solid‐state optical materials.

## Graphical abstract

Various machine‐learning methods and molecular descriptors are used for the prediction of molecular optical properties upon aggregation (aggregation‐induced emission (AIE)/aggregation‐caused quenching (ACQ)). The proposed multi‐modal ensemble method achieves the best and most robust performance (93.8% accuracy). The reliability of prediction is verified by several newly designed molecules that are structurally distant from known compounds.

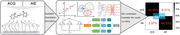

## 1 Introduction

Molecules are often used as films or aggregates in practical applications. As such, it is crucial to predict the performance of molecules when forming aggregates. Aggregation‐induced emission (AIE) is a concept first coined in 2001 describing the unusual emission enhancement of molecular species upon aggregate formation.^[^ ^1^, ^2^ ^]^ Since then, a new requisite research area of AIE has been opened to scientists and engineers and luminogens with AIE behavior are termed as AIEgens.^[^ ^3^, ^4^, ^5^, ^6^ ^]^ In the past decade, the superior property of AIEgen has initiated many unprecedented scientific studies and innovated the design of luminescent materials.^[^ ^7^, ^8^, ^9^, ^10^, ^11^, ^12^, ^13^ ^]^ For example, AIEgen‐based nanoparticles could be designed to show higher brightness and better stability than quantum dots;^[^ ^4^, ^14^, ^15^ ^]^ the device performance of non‐doped organic light‐emitting diodes can be significantly improved through the incorporation of AIE property into thermally activated delayed fluorescence emitters.^[^ ^16^, ^17^ ^]^ Other examples include various stimulus‐responsive luminescent materials.^[^ ^7^, ^8^, ^18^ ^]^ The high brightness of AIEgens in the solid state makes their responses more visible to the naked eye. Besides, based on AIEgens with suppressed non‐radiative decay in the aggregate state, molecular engineering has yielded AIE photosensitizers (PSs) with higher brightness and better photosensitization in nanoparticles as compared to that for traditional PSs.^[^ ^9^, ^19^ ^]^

The uniqueness and versatility of AIEgens increase their demand in a wide range of fields.^[^ ^20^, ^21^, ^22^, ^23^ ^]^ Much effort has been devoted to the design and synthesis of new AIEgens.^[^ ^24^, ^25^ ^]^ The key to designing a new AIEgen is the prediction of the AIE property from its molecular structure, which requires a high level of structure–property understanding of the AIE phenomenon.^[^ ^26^, ^27^, ^28^ ^]^ However, owing to the diverse impact factors in the process of molecular aggregation, precise prediction of AIE property from molecular structure remains challenging. Moreover, the complexed AIE mechanisms are based on various dimensions of photophysics, for example, restriction of intramolecular rotation or vibration,^[^ ^29^ ^]^ restriction of excited‐state deformation,^[^ ^30^ ^]^ suppression of Kasha's rule,^[^ ^31^ ^]^ and et al., making it difficult to predict the AIE property in unknown molecular space. As a result, many AIEgens were designed by trial and error, which make the design of next generation AIEgens difficult and slow. In fact, a majority of AIEgens is derived from a few AIE cores like tetraphenylethylene (TPE), triphenyl amine (TPA), and tetraphenyl pyran (TPP),^[^ ^1^, ^26^ ^]^ limiting the performance optimization in a particular application.

Furthermore, more and more AIEgens with unclear mechanisms have been reported,^[^ ^30^, ^32^ ^]^ implying an urgent demand to improve the current understanding of AIE.

Machine learning (ML) techniques have been rising in popularity and found inspiring success in property prediction in various domains such as drugs, energy, and catalysis materials.^[^ ^33^, ^34^, ^35^, ^36^ ^]^ By scanning a large dataset and extracting relationships from molecular features that are required, ML can predict a wide range of properties without a substantial fundamental understanding of the underlying chemistry or physics behind these.^[^ ^37^, ^38^, ^39^ ^]^ When fundamentals are well‐understood, ML might provide additional scientific insights of different dimensions, thus enhancing chemical intuition and enriching design strategies.^[^ ^40^, ^41^ ^]^ Considering the multiple factors of AIE phenomenon and the complexity of mechanisms behind, ML is very likely to improve the current understanding of AIE and contribute to the prediction of AIE property. With the precise prediction of AIE property via an artificial intelligence (AI) system, even unexperienced AIE researchers will be capable of designing a molecular structure with AIE properties in an unknown molecular space.

**Figure 1:**
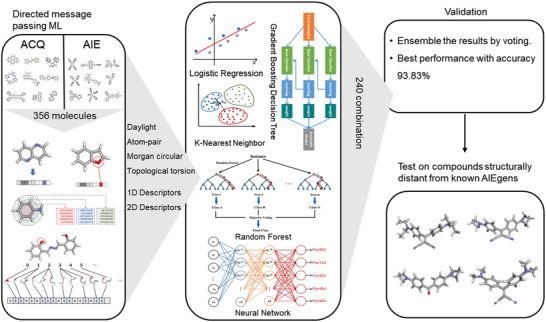
Flowchart of the proposed machine learning (ML)‐assisted prediction of AIE/ACQ properties and experimental validation of newly designed molecules.

In this work, we established a database containing 356 experimentally tested AIE/aggregation caused quenching (ACQ) molecules collected from the literature. We first studied the representation of molecules, that is, the process of generating embedding vectors. This is the most important step in molecular ML as better embedding vectors can compute and predict the molecules more effectively.^[^ ^42^, ^43^ ^]^ Here we consider two models of molecular expression, including qualitative and quantitative molecular descriptors. The most commonly used qualitative molecular descriptors are molecular fingerprint descriptors. We used Morgan circular fingerprint, Daylight fingerprint, atom‐pair fingerprint, and topological torsion fingerprint to generate embedding vectors, respectively. Herein, five ML algorithms, that is, logistic regression, K‐nearest neighbor (KNN), gradient boost, random forest, and neural network,^[^ ^44^, ^45^, ^46^ ^]^ were applied on different embedding vectors (**Figure** **1**). The experimental results show that ML methods can well predict AIE molecules. In order to build a more robust model, we adopted an integrated voting strategy to determine the final molecular prediction category. Through this strategy, the model can not only learn molecular information of the current data set, but also predict the combination information of unknown molecules. Last, we independently verified our proposed strategy of models by synthesizing three new molecules. The predictions of the model were in good agreement with the experimental results. Through this work, we set up a new methodology for AIE research, that is, prescreening the designed AIE molecules by ML models and then only focusing on those that pass the ML virtual assessment in subsequent experiments. This approach will accelerate the process of developing high‐performance AIEgens.

## 2 Results and Discussion

There are hundreds of AIEgens reported in the literature. When collecting data, we screened the molecules according to the following criteria: 1) capture representative AIE/ACQ counterparts that show similar molecular structures but opposite AIE/ACQ property, for example, a molecule's shift from ACQ to AIE by replacing its atoms, or increasing chain length, or changing the conjugated structure or position of connection (Figures S1 and S2, Supporting Information) screen molecules derived from the same AIE core, especially the classic cores. To avoid an unbalanced and biased dataset, we restricted the number of molecules derived from the same core. At last, 356 molecules were collected for model training.

Next, we present experimental analysis to demonstrate the effectiveness of the methods. We first employ molecular descriptors to characterize molecules, which is a key issue in molecular ML. In this work, two categories of molecular descriptors are used as different modes to predict molecular properties, that is, quantitative descriptors and qualitative descriptors, respectively.^[^ ^47^, ^48^, ^49^ ^]^ By this way, our approach can take into account both quantitative and qualitative properties of the molecules. Then, five popular ML methods are proposed to predict whether a molecule has AIE properties. These methods include logistic regression (LR), KNN, gradient boost (GB), random forest (RF), and multilayer perception (MLP) as a class of neural network. We also propose a strategy to integrate the results of different methods and modes and use the integrated results as the final predictive output. In order to compare and evaluate the effectiveness of the algorithms, we employed some evaluation metrics including accuracy, area under the curve (AUC) and F1‐score. In our experiment, tenfold cross‐validation was used to evaluate different methods with different descriptors. We used fivefold cross‐validation on the training set to select the hyperparameters. The detailed descriptions are available in the Supporting Information.

The results of separate tests for different methods based on qualitative and quantitative descriptors are shown in **Table** **1**. Here we refer to this strategy as single‐modal training. Qualitative descriptors consider five fingerprints and encode 2048 bits in length. Quantitative descriptors use 108‐dimensional features, including 1D and 2D shown in Table S1, Supporting Information. In addition to the five baseline methods described above, we also adopt a voting strategy, named as the ensemble method. Considering all methods and all modes, a total of 25 different combinations are adopted. Each combination votes on the classification results, and the category with more votes is the final result. This avoids the misclassification caused by under‐fitting or over‐fitting in individual combinations and enhances the decision making for each method. Experimental results show that the ensemble voting method is more robust than a single classification model. Note that the best result of each method with different fingerprints is marked in bold.

**Table 1:** Prediction performance results of qualitative and quantitative descriptors of baseline and ensemble methods based on single‐modal strategy

Methods	Train accuracy	Test accuracy	AUC	F1‐score
Logistic regression				
Morgan	0.9791 ± 0.0264	0.9160 ± 0.0415	0.9100 ± 0.0449	0.9158 ± 0.0416
Daylight	**0.9922 ± 0.0151**	**0.9329 ± 0.0280**	**0.9292 ± 0.0227**	**0.9329 ± 0.0274**
Atom‐pair	0.9778 ± 0.0299	0.9103 ± 0.0514	0.9090 ± 0.0550	0.9107 ± 0.0512
Topological	0.9332 ± 0.0053	0.9244 ± 0.0374	0.9181 ± 0.0438	0.9242 ± 0.0376
Quantitative descriptors	0.6236 ± 0.0038	0.6237 ± 0.0340	0.5000 ± 0.0000	0.4797 ± 0.0419
K‐nearest neighbor				
Morgan	0.9257 ± 0.0190	0.8879 ± 0.0430	0.8729 ± 0.0474	0.8866 ± 0.0442
Daylight	**0.9276 ± 0.0153**	0.8963 ± 0.0545	0.8827 ± 0.0639	0.8952 ± 0.0559
Atom‐pair	0.9151 ± 0.0071	0.9048 ± 0.0469	**0.9072 ± 0.0462**	0.9056 ± 0.0465
Topological	0.9154 ± 0.0051	**0.9075 ± 0.0472**	0.9058 ± 0.0502	**0.9077 ± 0.0471**
Quantitative descriptors	0.9017 ± 0.0133	0.8740 ± 0.0684	0.8716 ± 0.0726	0.8748 ± 0.0675
Gradient boost				
Morgan	0.8914 ± 0.0156	0.8851 ± 0.0577	0.8818 ± 0.0571	0.8852 ± 0.0575
Daylight	**0.9822 ± 0.0151**	0.9017 ± 0.0461	0.8911 ± 0.0538	0.9011 ± 0.0468
Atom‐pair	0.9194 ± 0.1000	0.8713 ± 0.0905	0.8602 ± 0.1298	0.8579 ± 0.1287
Topological	0.8814 ± 0.0507	0.8795 ± 0.0711	0.8746 ± 0.0709	0.8792 ± 0.0711
Quantitative descriptors	0.9813 ± 0.0329	**0.9189 ± 0.0419**	**0.9182 ± 0.0452**	**0.9192 ± 0.0417**
Random forest				
Morgan	0.9878 ± 0.0047	0.9074 ± 0.0281	0.9017 ± 0.0323	0.9071 ± 0.0284
Daylight	0.9919 ± 0.0040	0.9213 ± 0.0433	0.9080 ± 0.0528	0.9204 ± 0.0444
Atom‐pair	0.9913 ± 0.0125	0.9271 ± 0.0402	0.9315 ± 0.0412	0.9276 ± 0.0400
Topological	0.9953 ± 0.0067	**0.9329 ± 0.0397**	0.9283 ± 0.0464	**0.9326 ± 0.0398**
Quantitative descriptors	**0.9956 ± 0.0060** *	0.9326 ± 0.0475	**0.9338 ± 0.0561** *	0.9325 ± 0.0482
MLPClassifier				
Morgan	0.9813 ± 0.0226	0.9159 ± 0.0414	0.9088 ± 0.0443	0.9156 ± 0.0418
Daylight	**0.9885 ± 0.0074**	**0.9385 ± 0.0298** *	**0.9332 ± 0.0359**	**0.9383 ± 0.0299** *
Atom‐pair	0.9784 ± 0.0296	0.8910 ± 0.0808	0.8838 ± 0.0936	0.8884 ± 0.0869
Topological	0.9420 ± 0.0046	0.9217 ± 0.0347	0.9170 ± 0.0383	0.9216 ± 0.0347
Quantitative descriptors	0.8394 ± 0.1154	0.8396 ± 0.0923	0.8228 ± 0.1482	0.8199 ± 0.1320
Ensemble	—	0.9274 ± 0.0416	0.9226 ± 0.0444	0.9273 ± 0.0416

* Superscript symbol * indicates optimal results for all methods.

From the results, we can see that the train accuracy and AUC of the random forest based on the quantitative descriptors are better than others. The MLP Classifier method based on Daylight fingerprint has the best performance in test accuracy and F1‐score. This shows that the two types of descriptors (two modals) are both important for the prediction performance. logistic regression has poor performance based on quantitative descriptors, which indicates that the quantitative descriptors are not suitable for linear fitting. Therefore, the nonlinear method can yield better results. Random forest has relatively good results on all fingerprints, because it can handle high‐dimensional data, and for unbalanced data sets, it can balance the errors. Gradient boost is an ensemble method based on the decision tree, however, its performance is not as good as the random forest, is probably due to overfitting. In the multi‐modal experiment, the potential overfitting is more obvious. The result of MLP Classifier is next to the random forest. Neural network is the algorithm that receives the most attention at present, which, however, needs more data points for training to get better results. For a suitable fingerprint, the training result of Daylight fingerprint is better than other descriptors in stability. This is because the topological mode of the Daylight fingerprint is more in line with the molecular structures of AIEgens.

In our data set, 134 samples have AIE properties while 222 samples have ACQ properties, which is a slightly unbalanced data set. Determining the category of hard samples is the key problem. Hard samples are samples close to the classification plane, and in general, these samples are also data that are not adequately obtained and mined in the data set. The intra‐class distance of these data tends to be larger and may be closer to the inter‐class samples. In other words, these samples are relatively rare samples in this experiment. The biggest contribution of AIE molecular prediction to the future is to predict data that has not been generated in the current experiment, that is, data that has not been seen ever. In this way, researchers can better guide the experimental generation of data.

The ensemble method is proposed to explore the classification plane and the prediction of hard samples. In order to illustrate the effectiveness of the proposed ensemble method, the confusion matrix of the benchmarking methods are shown in Figure S6, Supporting Information. As seen from the figures, the most accurate classifier is the MLP classifier on Daylight fingerprint. There are 22 misclassification samples, among which 11 molecules with AIE properties are misclassified as ACQ and 11 molecules with ACQ properties are misclassified as AIE instead. The next most accurate classifier is the random forest on quantitative descriptors. There are 24 misclassified samples, 8 AIE are misclassified as ACQ, and 16 ACQ are misclassified as AIE. There are 26 misclassification samples by ensemble method, 13 AIE are misclassified as ACQ and 13 ACQ are misclassified as AIE. This may be caused by unsatisfactory results for certain methods used in single‐modal, such as linear regression. Therefore, there is a big gap between the classification planes of different methods, and it is difficult to select one method as the final result.

**Figure 2:**
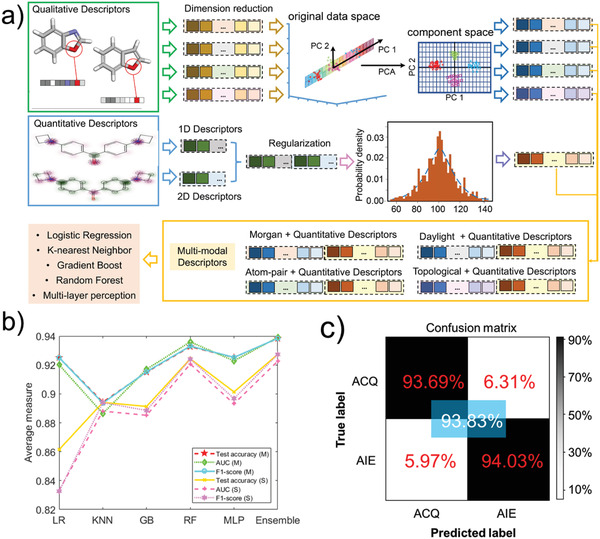
a) Schematic illustration of multi‐modal descriptors. b) Average results of different methods. c) Confusion matrix of ensemble method based on multi‐modal descriptors.

**Table** **2** shows the results of different methods based on the multi‐modal strategy. We also present the average results of single‐modal and multi‐modal on five different methods and confounding matrix of ensemble method base on multi‐modal descriptors in **Figure** **2a**. Multi‐modality here refers to combining two types of descriptors into a feature vector for training, which is shown in Figure 2b. We first performed dimensionality reduction using principal component analysis (PCA) on the 2048 dimensional fingerprint feature. The aim was to make fingerprint and quantitative descriptor have a closer dimension and avoid focusing on one modal. After this process, 356 dimensional features are obtained, but all information of the original data is retained. The specific processes and principles are described in the Supporting Information. In addition, we made a *z*‐score standardization for the quantitative descriptors, so that they have the same scale with PCA dimensionless data. Therefore, for multi‐modal data, the feature dimension is 464 (356 + 108).

**Table 2:** Prediction performance results of baseline and ensemble methods based on multi‐modal strategy

Methods	Train accuracy	Test accuracy	AUC	F1‐score
Logistic regression				
Morgan + Quantitative	0.9863 ± 0.0071	0.9215 ± 0.0323	0.9130 ± 0.0354	0.9211 ± 0.0323
Daylight + Quantitative	**0.9925 ± 0.0085**	**0.9356 ± 0.0277**	**0.9293 ± 0.0291**	**0.9355 ± 0.0272**
Atom‐pair + Quantitative	0.9791 ± 0.0289	0.9217 ± 0.0427	0.9219 ± 0.0441	0.9221 ± 0.0426
Topological + Quantitative	0.9828 ± 0.0117	0.9217 ± 0.0427	0.9163 ± 0.0476	0.9215 ± 0.0427
K‐nearest neighbor				
Morgan + Quantitative	0.9185 ± 0.0200	0.8795 ± 0.0545	0.8660 ± 0.0661	0.8775 ± 0.0573
Daylight + Quantitative	**0.9294 ± 0.0138**	0.8906 ± 0.0441	0.8755 ± 0.0535	0.8893 ± 0.0455
Atom‐pair + Quantitative	0.9235 ± 0.0131	**0.9133 ± 0.0439**	**0.9117 ± 0.0461**	**0.9136 ± 0.0437**
Topological + Quantitative	0.9089 ± 0.0069	0.8936 ± 0.0557	0.8924 ± 0.0600	0.8941 ± 0.0553
Gradient boost				
Morgan + Quantitative	1.0000 ± 0.0000*	**0.9302 ± 0.0416**	**0.9343 ± 0.0406**	**0.9308 ± 0.0410**
Daylight + Quantitative	1.0000 ± 0.0000*	0.9157 ± 0.0360	0.9147 ± 0.0453	0.916 ± 0.0369
Atom‐pair + Quantitative	1.0000 ± 0.0000*	0.9271 ± 0.0359	0.9261 ± 0.0392	0.9271 ± 0.0360
Topological + Quantitative	0.9483 ± 0.1551	0.8873 ± 0.1108	0.8931 ± 0.1011	0.8875 ± 0.1109
Random forest				
Morgan + Quantitative	0.9981 ± 0.0032	0.9410 ± 0.0411	0.9418 ± 0.0465	0.9411 ± 0.0412
Daylight + Quantitative	0.9981 ± 0.0032	0.9440 ± 0.0304*	0.9499 ± 0.0288*	0.9445 ± 0.0300*
Atom‐pair + Quantitative	**0.9997 ± 0.0009**	0.9187 ± 0.0462	0.9214 ± 0.0509	0.9191 ± 0.0462
Topological + Quantitative	0.9975 ± 0.0027	0.9271 ± 0.0422	0.9309 ± 0.0467	0.9275 ± 0.0422
MLPClassifier				
Morgan + Quantitative	**0.9991 ± 0.0020**	0.9216 ± 0.0388	0.9154 ± 0.0397	0.9214 ± 0.0387
Daylight + Quantitative	0.9966 ± 0.0083	**0.9413 ± 0.0259**	**0.9369 ± 0.0244**	**0.9412 ± 0.0256**
Atom‐pair + Quantitative	0.9984 ± 0.0038	0.9188 ± 0.0420	0.9217 ± 0.0452	0.9192 ± 0.0420
Topological + Quantitative	0.9959 ± 0.0063	0.9190 ± 0.0502	0.9174 ± 0.0477	0.9192 ± 0.0494
Ensemble	—	0.9383 ± 0.0376	0.9391 ± 0.0445	0.9384 ± 0.0379

It can be seen from the results that the multi‐modal prediction is generally better than single‐modal prediction. Among them, the results of logistic regression were relatively close, because the fitting of features by linear regression method was limited. When the data is nonlinear, it is difficult to achieve greater improvement, as well as dealing with the problem of data imbalance. There was no significant improvement in the results of KNN. This is because the distance‐based KNN method has dimensionality disaster problem, and the prediction accuracy of minority categories is low. The results of gradient boost, random forest, and MLPClassifier have been improved significantly, because the feature with rich information plays a greater role in the complex method. The gradient boost obtained completely correct results on the three training feature sets, but the result on the test set was not as good as the random forest, which further indicated that the gradient boost had overfitting on the training set. random forest achieved the best performance in the test set on Daylight + quantitative descriptors. This shows that the fusion of the two modal features improves the separability of the classification space. The result of MLP Classifier is the best in the Daylight fingerprint, which is similar to the single‐modal result. It can be seen that the feature encoding method of Daylight fingerprint is more suitable for our data. Compared with the single modal, the ensemble method with multi‐modal data has obviously been improved and is closer to the optimal baseline method, which indicates that the multi‐modal data can correctly divide the classification plane for all the methods. In this case, the ensemble method can more effectively correct the misclassification of hard samples, and at the same time guide test of unknown samples more accurately.

**Figure 3:**
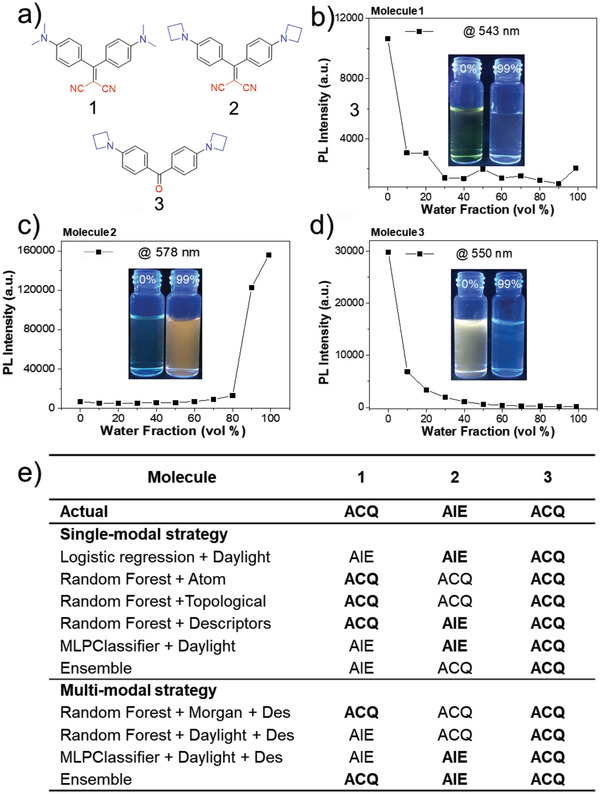
a) Structures of compound **1**–**3**. Plots of the maximum photoluminescence (PL) intensity of compound **1**–**3**, b–d) against water fractions (vol%). The inset shows photographs of the compounds in 0 and 99 vol% water under UV light (365 nm) illumination. e) Results of the different state‐of‐the‐art methods compared with ensemble strategy.

To validate the ability of our model in predicting new molecular structures, we designed a series of potential AIEgens with structures unlike any reported ones. The molecular structures of **1**–**3** are shown in **Figure** **3a**. In contrast to compound **1**, compound **2** possesses an additional carbon in the amino substituent structure, yielding a three‐membered ring. According to the known AIE mechanisms, compounds **1** and **2** are supposed to show similar AIE/ACQ property. However, our model predicts that compound **1** is ACQ while **2** is AIE. To validate the prediction, compounds **1** and **2** were prepared by Knoevenagel condensation and Buchwald–Hartwig coupling followed by AIE characteristics study. The synthesis and characterization are described in the Supporting Information. The AIE characteristics of all the compounds were studied by monitoring their emission properties in DMSO/water mixtures with different volume fractions of water. The titration results of **1** and **2** are shown in Figure 3b. Compounds **1** and **2** show ACQ and AIE properties, respectively, consistent with the ML prediction. Next, we replaced the dicyano group of **2** with a carbonyl group and obtained compound **3**. From the results of **1** and **2**, it is obvious that the three‐membered amino ring of **2** plays a crucial role in its AIE behavior and thus **3** is supposed to be AIE active as well. However, our ML model predicts that compound **3** is an ACQ molecule. To validate the prediction, we synthesized **3** followed by the same AIE characteristics study and the result shows that **3** is ACQ, indicating the excellent accuracy of our model in predicting new structures superior to human perception. Figure 3e shows the results of the different state‐of‐the‐art methods. These methods are competing methods with the ensemble model which we have compared in the last section. It can be seen from the table that only the prediction results of multi‐modal ensemble and single‐modal random forest are completely correct. However, from Figure 3e, the results of single‐modal random forest on the test set are not as good as that of the multi‐modal ensemble. This demonstrates that multi‐modal ensemble is more robust, which means that for different data types, including known and unknown data, it has both predictive and exploratory capabilities.

## 3 Conclusion

On the basis of a database containing AIE/ACQ molecules collected from the literature, various programming language expressions of small molecules including Morgan circular fingerprint, Daylight fingerprint, atom‐pair fingerprint, topological torsion fingerprint, and quantitative descriptors were used to build ML models to predict the AIE/ACQ properties of different fluorophores. The proposed multi‐modal ensemble method achieves the best and most robust performance. This strategy considers the properties of multiple descriptors and combines the inference advantages of multiple methods. Therefore, it can not only learn the existing molecular structure, but also has the ability to predict an unknown structure.

The reliability of our ML model in predicting brand new molecules has been further demonstrated. We compared the prediction from the ML models and the results of the experiment for three newly designed small molecules. The ML predictions are consistent with experimental results. The limitation of current model is its applicability in non‐rotor structures as most considered AIEgens in our dataset are rotor structure based. We have developed a scheme to help AIEgen design by combining ML approaches and experimental analysis. That is, a large number of AIEgens could be screened through a pre‐evaluation and classification ML model, and then the identified candidates will be synthesized and further tested by experiments. Our study on the relationship between the chemical structure of molecule and AIE/ACQ prediction could speed up new AIEgen design and hence accelerate the development of high‐performance organic luminescent materials.

## 4 Experimental Section

### Molecular Descriptor

Molecular characterization, that is, how to encode molecular structures, is a key issue in molecular machine learning. The complexity of molecular characterization is a numerical description comparing the similarities between two compounds.^[^ ^1^, ^2^ ^]^ In order to make molecular comparisons computationally easier, the structure needs to be simplified and abstract. Quantitative structure–activity relationship (QSAR) is an important tool for molecular characterization. It uses mathematical statistics to explain the quantitative change rule between the activity or physical and chemical characteristics of a compound and its molecular structure.

Molecular descriptors are the primary way QSAR is calculated. Accurate molecular descriptors play a decisive role in the reliability of molecular evaluation. A molecular descriptor is a measure of some aspect of a molecule, including its physical and chemical properties, or a numerical indicator derived from various algorithms. Currently, more than 5000 molecular descriptors are provided and calculated by various software. The RDkit is used to generate molecular descriptors as numerical descriptors^[^ ^8^ ^]^ for predictive experiments.

Molecular descriptors can be divided into quantitative descriptors and qualitative descriptors. Quantitative description includes descriptors based on molecular graph theory, various theoretical or experimental spectral data (such as ultraviolet spectrum), molecular composition (such as number of hydrogen bond donors, number of chemical bonds), physical and chemical properties (such as ester water distribution coefficient), molecular field descriptors, and molecular shape descriptors, etc. Qualitative descriptors are commonly referred to as molecular fingerprints, which represent the structure, properties, fragments or substructures of molecules with some kind of code. In this paper, these two molecular descriptors were used as different modes to predict molecular properties. So this approach takes into account both the quantitative and qualitative properties of the molecules.

### Qualitative Descriptors

Molecular fingerprinting is by far the most popular way of quantitative descriptors, which converts a molecule into (encodes) a series of binary digits (bits) to indicate the presence or absence of a specific substructure in the molecule (see Figure S1, Supporting Information). Each molecule first applies a hash function and then generates a fingerprint based on the feature. Comparing fingerprints allows you to determine similarities between two molecules, find matching query substructures, and so on. By doing so, statistical analysis and/or machine‐learning techniques on this group of molecules to gain new insights that were not available to humans could be performed. Molecular classification, for example, reduces the similarity between molecules of the same class (intra‐class) and increases the similarity between molecules of the different class (inter‐class).

It is difficult to compare molecules directly in structure, so the comparison between molecules needs to be quantified first. The quantized molecules are represented in the form of bit strings, and each bit corresponds to a segment of a molecule (Figure S2, Supporting Information). Similar molecules should have many pieces in common, and this should be expressed numerically as a similar vector. In this way, the similarity calculation method based on distance, divergence, and correlation coefficient can be easily applied to molecules. There are many molecular fingerprint methods, the most commonly used are Morgan circular fingerprint, Daylight fingerprint, atomic pair fingerprint, and topological twist fingerprint.

The extended connectivity fingerprint (ECFP) is a molecular fingerprint that can represent the internal structure of a compound, which is derived from the Morgan algorithm.^[^ ^9^ ^]^ In recent years, ECFP has become the industry standard method for circular molecular fingerprint, which is often used as a benchmark to compare the effect of new methods in machine learning. Morgan circular fingerprint (MCP) is part of the family of ECFP, using Morgan^[^ ^10^ ^]^ generation algorithm. MCP searches substructures of all given steps in the compound through Morgan search algorithm, and then obtains the hash value of each substructure through hash, thus forming the corresponding fingerprint. When this fingerprint is used, it will produce a fingerprint of variable length according to different set diameters. They record each environment from the atom up to a specified radius (see Figure S3, Supporting Information). MCP can determine the absence or presence of molecular function and is widely used in similarity search of complete structures.

Topological or path‐based fingerprint starts with an atom, intercepts molecular fragments along the path to a specified length, and hashed each fragment to get a fingerprint (see Figure S4, Supporting Information). This fingerprint can adjust the path length, calculate all the subgraphs between the minimum path and the maximum path, so it is suitable for arbitrary molecules, and can quickly search for substructures and filter molecules. The Daylight fingerprint is the most prominent representative of this kind of fingerprint. The fingerprint generator produces a fingerprint similar to that produced using the Daylight fingerprint algorithm.^[^ ^12^, ^13^ ^]^ Atom‐pair fingerprint and topological torsion fingerprint are also two common topological fingerprints. Atom‐pair fingerprint identifies each atom in the molecule as the shortest path based on the environment. Meanwhile topological torsion fingerprints are generated by constructing a topological dual angle descriptor using the bonding paths of four non‐hydrogen atoms. The two fingerprint algorithms are similar in that they contain information in three dimensions: atomic number, number of *π* electrons, and number of adjacent atoms. In addition, they can all be expressed in sparse form and explicit form, and you can view the chemical information represented by the fingerprint.

### Quantitative Descriptors

Quantitative descriptors can be divided into 1D, 2D, and 3D, etc. according to the calculation of molecular structure dimensions required. RDKit provides many methods for calculating descriptors, which can be used for molecular screening, drugenicity assessment, etc. Here we filter 108 1D and 2D descriptors to quantify features, including 201D and 88 2D molecular descriptors (see Table 1 for details).

### Logistic Regression

Logistic regression^[^ ^3^ ^]^ is called “regression” because the basic idea is based on regression. However, it is used to deal with classification problems, mainly for binary classification, that is, there are only two outputs, representing two categories. Here, let *X* denote the samples, with corresponding outputs *Y*. Θ is the covariant matrix. The classification label *Y* ∈ {0, 1}, where 1 represents positive samples and 0 represents negative ones, was assumed. In logistic regression, we find a hypothetical function *h*(*x*) = *g*(*θ* ^*T*^ *x*) based on the prediction of the actual value of the linear function *θ* ^*T*^ *x* output, and map the actual value to a value between 0 and 1. If *h*(*x*) ≥ 0.5 then *y* = 1, which would give a prediction of a positive sample. And if *h*(*x*) < 0.5, then *y* = 0, which would give a prediction of a negative one. The sigmoid function was selected as the activation function in logistic regression, which is defined as:

(1) $$g\left(z\right)=\frac{1}{1+e^{\left(-z\right)}}$$

Then the predictive function of logistic regression output is:

(2) $$h_{\theta }\left(x\right)=g\left(\theta^{T}x\right)=\frac{1}{1+e^{\left(-\theta^{T}x\right)}}$$

Logistic regression is a logarithmic probability of a linear combination of features to fit the probability of a true label as a positive example $\left(\ln\frac{y}{1-y}=\theta^{T}x+b\right)$. In other words, the probability that the input *x* is classified to be category 1 and category 0 is:

(3) $$\begin{array}{c} P\left(y=1|x;\theta \right)=h_{\theta }\left(x\right)\\ P\left(y=0|x;\theta \right)=1-h_{\theta }\left(x\right) \end{array}$$

### K‐Nearest Neighbor

KNN^[^ ^4^ ^]^ is a relatively mature pattern recognition algorithm and one of the simplest classification algorithms. Considering the *k* closest samples of a data point, if most of the samples belong to a certain category, the data point also belongs to this category. Two key factors that affect KNN were the number of neighbors *k* and the calculation of distance. *k* was usually an integer not greater than 20 and the distance was generally using Euclidean distance. The Euclidean distance is defined as $d=\sqrt{\sum_{i=0}^{n}{\left(x_{i}-y_{i}\right)}^{2}}$, where *n* is the number of samples.

The neighbors selected in the KNN algorithm were all objects that have been correctly classified. This method determined the category to which the sample to be classified belongs only based on the category of the nearest sample or samples. Therefore, the KNN algorithm process could be described as: 1) calculate the distance between test data and each training data; 2) sort by increasing distance; 3) select the K points with the smallest distance; 4) determine the occurrence frequency of the category of the first K points; 5) return the category with the highest frequency in the first K points as the predicted classification of test data.

### Gradient Boosting Decision Tree

Gradient boosting decision tree (GBDT)^[^ ^5^ ^]^ is a very popular model in machine‐learning applications with excellent performance. It is a representative algorithm in boosting series.^[^ ^18^, ^19^ ^]^ Boosting is a progressive model combination method. Each new classifier improves on the prediction result of the previous classifier. Therefore, boosting is a model combination method that reduces bias. GBDT is an iterative decision tree algorithm, which is composed of multiple decision trees. The conclusions of all trees are summed up as the final answer, and the integration method is gradient boosting. The intuitive understanding was: each round of predictions had residuals with actual values, and the next round of predictions was performed based on the residuals, and finally all predictions were added together to obtain the result. GBDT through multiple rounds of iteration, each iteration generated a weak classifier, each classifier was trained based on the residuals of the previous classifier. Since the training process was to reduce the deviation to continuously improve the accuracy of the final classifier. The requirements for weak classifiers were generally simple enough and have low variance and high bias. The weak classifier was generally selected as the CART TREE^[^ ^19^ ^]^ (the classification regression TREE). Due to the high bias and simple requirements, the depth of each classification regression tree was not very deep. The final total classifier was a weighted summation of the weak classifiers obtained in each round of training.

Using a decision tree to represent the basic model of GBDT, then GBDT can be expressed as:

(4) $$f_{M}\left(x\right)=\sum_{m=1}^{M}T(x;\Theta_{m})$$

where *T*(*x*; Θ_m_) represents the decision tree. *M* is the number of trees. The forward distribution algorithm was adopted to first determine the initial boosting tree *f*_o_(*x*) *=* 0. Then the model in step *m* was:

(5) $${\hat{\Theta }}_{m}=\arg\;\underset{}{}\min_{\Theta_{m}}\sum_{i=1}^{N}L\left(y_{i},f_{\left(m-1\right)}\left(x_{i}\right)+T\left(x_{i};\Theta_{m}\right)\right)$$

where *L*() is the loss function. The loss function selected by the regression algorithm was generally the mean square error (least squares) or the absolute value error. In the classification algorithm, the logarithmic function was used to represent the loss function.

### Random Forest

Random forest^[^ ^6^ ^]^ is a flexible and convenient machine‐learning algorithm, which is an ensemble learning algorithm based on decision tree. The idea of ensemble learning is to solve the inherent defects of a single model or a group of models, so as to integrate more models, learn from each other, and avoid limitations. Bagging and boosting are two main algorithms of ensemble learning. Both bagging and boosting combine existing classification or regression algorithms in a certain way to form a more powerful classifier. This is an assembly method of classification algorithm, which assembles weak classifier into strong classifier. Bagging comes from bootstrap aggregating, which means self‐service sampling ensemble. This method divides the training set into *k* new training sets, and then builds a model on each new training set, which is irrelevant. During the prediction, the *k* models are classified by voting to get the classification results. For the regression problem, the mean value of *k* models is calculated as the final result.

Random forest is a special bagging method that uses a decision tree as a model in bagging. Decision tree divides the space with a hyperplane, each time splitting the current space into two. This makes each leaf node a disjoint region in space. When making a decision, the input sample goes down step by step according to the value of each feature and falls into one of the *N* regions (assuming *N* leaf nodes). Therefore, the workflow of random forest could be summarized as follows:

i) *k* features were randomly selected from the data set, with a total of *m* features (where *k* was less than or equal to *m*). Then a decision tree was built based on these *k* features;
ii) Repeat *n* times, and these *k* properties were randomly combined to create *n* decision trees;
iii) Random samples were input for each decision tree to predict the results, and *n* results could be obtained from *n* decision trees;
iv) The votes of each predicted target were calculated, and the predicted target with the highest number of votes were taken as the final prediction of the random forest.

### Neural Network

Artificial neural network (ANN)^[^ ^7^ ^]^ is a computational nonlinear model widely used in machine learning. It consists of three parts, the input layer, the hidden layer, and the output layer. Each layer contains a certain number of neurons. Input layer data is passively received through the node and then passed to subsequent hidden layers. The hidden layer processes the data using various mathematical functions, which are then passed to subsequent output layers. The final output layer provides the final solution for the network.

Perceptron or single‐layer neural network is the simplest neural computing model. The perceptron can be regarded as a single neuron with input *x*_1_, *x*_2_, *x*_3_, …, *x_N_*, and output *y* = *f*(*x*). Each perceptron input has the weight *w*_1_, *w*_2_, *w*_3_, …, *w_N_*. If a weight is less than 1, it will weaken the input. If it is greater than 1, it will enlarge the input. In addition, there is an input 1 in the perceptron model, with a fixed weight *b*, called deviation, and used as the target value for training perceptron. Thus, the output of the perceptron can be expressed as follows:

(6) $$f\left(x\right)=f\left(\sum_{i}^{M}\omega_{i}x_{i}+b\right)$$

where *f*(*x*) is called the activation function or transfer function and was used to determine the value of the perceptron output. Common activation functions include sigmoid function, tanh function, and ReLU function.

### Principal Component Analysis

PCA is the most widely used data dimension reduction algorithm, which can be used to extract the main characteristic components of data. PCA expresses data by using a small number of features that contains the core information of the data, and finds a few features that describe the key information. The idea of PCA is based on the theory of maximum variance, which maps *n*‐dimensional features to *k*‐dimensions *(k < n)*. This *k*‐dimension is new orthogonal features called principal component. Generally used in scenarios where the original data have many features and features have obvious correlations. Due to PCA only selects the most critical information, it has better resistance to interference. In this work, the default value for the number of principal components (number of preserved features) in the input parameter of PCA, which was defined as the principal components = min (number of samples, number of features) was used. Therefore, the number of features retained in this experiment according to the dataset was the number of samples, which had 356 dimensional features.

The PCA member parameter explained variance_ratio, was outputted which indicated the ratio of the variance of each principal component to the total variance after dimensionality reduction. The larger the ratio, the more important the principal component. After summing the explained variance_ratio, that is, the ratio of all retained features to the total variance, the result was always 1 in these experiments. This means that the data was reduced from 2048 dimensions to 356 dimensions to retain all the information of the original data without loss of information.

### Metrics

A standard format for accuracy evaluation is the confusion matrix, also known as the error matrix. It visually describes the predictive performance of the model and compares it from four quadrants:

True positives (TP): Samples are predicted positive and actually are positive.

True negatives (TN): Samples are predicted negative and actually are negative.

False positives (FP): Samples are predicted positive but actually are negative.

False negatives (FN): Samples are predicted negative but actually are positive.

Based on the confusion matrix, the accuracy is defined as:

(7) $$\mathrm{Accuracy}\;=\frac{\mathrm{TP}\;+\;\mathrm{TN}}{\mathrm{TP}\;+\;\mathrm{TN}\;+\;\mathrm{FN}\;+\;\mathrm{FP}}\;$$

Accuracy is the most common evaluation metric. For binary classifiers, the model can be well evaluated in accuracy when classes are balanced. However, when the classes were unbalanced, the accuracy is partial to the majority class and has little effect on the evaluation of the minority classes. Therefore, two other comprehensive metrics, F1 and AUC were employed. F1‐score considered both precision and recall to find a balance between them:

(8) $$\mathrm{Precision}\;=\frac{\mathrm{TP}}{\mathrm{TP}\;+\;\mathrm{FP}}\;$$

(9) $$\mathrm{Recall}\;=\frac{\mathrm{TP}}{\mathrm{TP}\;+\;\mathrm{FN}}\;$$

(10) $$F1\;=\frac{2\;\times \;\mathrm{Precision}\;\times \;\mathrm{Recall}}{\mathrm{Precision}\;+\;\mathrm{Recall}}\;\;$$

It could be seen from the equations that recall is described based on the true label, and precision is relative to model prediction. In general, if the model was greedy and wanted to cover more samples, it was more likely to make mistakes. In this case, there would be high recall and low precision. If the model was conservative and only made a prediction for its very certain sample, the precision would be high, but the recall would be relatively low. F1 took these two factors into account, and the higher the value, the better the result.

In order to illustrate AUC, it was needed to introduce the concepts of true positive rate (TPR) and false positive rate (FPR). Both metrics were based on the conditional probability of the true label, and the true distribution of the label did not affect TPR and FPR. In other words, these two metrics would not be affected by imbalanced data.

(11) $$\mathrm{TPR}\;=\frac{\mathrm{TP}}{\mathrm{TP}\;+\;\mathrm{FN}}\;$$

(12) $$\mathrm{FPR}\;=\frac{\mathrm{TP}}{\mathrm{TN}\;+\;\mathrm{FP}}\;$$

where TPR is same as recall. The receiver operating characteristic curve (ROC curve) took FPR as the abscissa and TPR as the ordinate using different thresholds to draw the curve.

The area under curve (AUC) is the area under the ROC curve. Basically, the larger the AUC, or the curve is closer to the upper left corner (true positive rate = 1, false positive rate = 0), the better the model performance.

### Hyperparameters

Training data were split into five parts, four parts as sub‐training, and one portion as validation. The parameters with the best results were selected to train the final model. The hyperparameters were set as:

i) Logistic regression: the regularization parameter was chosen in the range [10^−3^,10^3^];
ii) KNN: the number of neighbors was chosen from [3, 5, 7, 9, 11, 13, 15, 17, 18];
iii) Gradient boosting: the minimum samples split and minimum samples leaf were in the range [0.1, 0.5], and the maximum depth was from values [3, 5, 7]; the subsample was chosen from [0.5, 0.75, 0.95];
iv) Random forest: the minimum samples split was in the range [2, 4], and the number of estimators was from [10, 50, 100, 200, 500];
v) Neural network: the learning rate search grid was in the range [10^−5^, 10^−1^] using a log‐scale; and the regularization parameter *α* was chosen from [10^−5^, 10^−3^] using a log‐scale.

### Confusion Matrix

The confusion matrix of ensemble method and competing method based on multi‐modal features are shown in Figure S6, Supporting Information. As seen, the highest accuracy rate was based on random forest on Daylight + Quantitative descriptors. There were 20 misclassified samples, of which 4 AIEs were misclassified as ACQ, and 16 ACQs were misclassified as AIE. Next to this method was MLPClassifier on Daylight + quantitative descriptors, with 21 misclassified samples, 11 AIE misclassified as ACQ, and 10 ACQ misclassified as AIE. First of all, both methods perform well in Daylight + Des features, which demonstrates the effectiveness of the fusion of these two features. Secondly, the results of the two methods were significantly different on the unbalanced classification plane. Random forest was fairer to a single unbalanced category, while MLPClassifier treated two unbalanced categories equally. This once again brings up the problem of evaluating the final result. The ensemble method had 22 misclassified samples, 8 AIEs were misclassified as ACQ, and 14 ACQs were misclassified as AIE. Although it was based on the results of the integrated voting of the 20 modes, it was also consistent with the results of balancing the two best methods. This indicated that the voting integration method could be predicted effectively and comprehensively, which was regarded as the final model.

## Conflict of Interest

The authors declare no conflict of interest.

## Supporting information

Supporting Information

## Data Availability

The code and data that support the findings of this study are openly available in GitHub at https://github.com/jiali1025/Prediction_of_Molecular_Optical_Properties.
