# Supplementary material for: Machine‐Learning‐Assisted Accurate Prediction of Molecular Optical Properties upon Aggregation
Source: Adv Sci (Weinh). 2021 Nov 25;9(2):2101074. doi: 10.1002/advs.202101074 (PMC8760175; doi:10.1002/advs.202101074)
Supplement: Supplementary file 1 — Supporting Information [file ADVS-9-2101074-s001.pdf]

WILEY-VCH

© 2021 Wiley-VCH GmbH

ADVANCED SCIENCE
Open Access

# Supporting Information

for *Adv. Sci.*, DOI: 10.1002/advs.202101074

# Machine Learning-Assisted Accurate Prediction of Molecular Optical Properties upon Aggregation

*Shidang Xu[+], Xiaoli Liu[+], Pengfei Cai, Jiali Li, Xiaonan Wang* and Bin Liu**

# Supporting Information

## Machine Learning-Assisted Accurate Prediction of Molecular Optical Properties upon Aggregation

*Shidang Xu[+], Xiaoli Liu[+], Pengfei Cai, Jiali Li, Xiaonan Wang [*] and Bin Liu[*]*

**Materials** Tetrahydrofuran (THF) was distilled from sodium benzophenone ketyl under dry nitrogen immediately prior to use. Azetidine, bis(4-(dimethylamino)phenyl)methanone, malononitrile, 4-bromoiodobenzene, and other chemicals and reagents for the synthesis were purchased from Sigma-Aldrich and Tee Hai Chem Ltd., and used as received without any further purification.

**Characterization.** NMR spectra were recorded on a Bruker ARX 400 NMR spectrometer. Chemical shifts are recorded in parts per million referenced according to residual solvent ($CDCl_3$ = 7.26 ppm) in $^1$H NMR and ($CDCl_3$ = 77.0 ppm) in $^{13}$C NMR. Mass spectra of synthetic small molecules were reported on the AmaZon X LC-MS for ESI. Mass spectra for proteomic study were recorded on a Finnigan LCQ mass spectrometer. UV-vis and photoluminescence spectra were recorded using Shimadzu UV-1700 and Perkin-Elmer LS 55 spectrometer, respectively. Hydrodynamic diameter and size distribution were tested by a Zetasizer Nano S (Malvern Instruments Ltd, Worcestershire, UK) at room temperature.

**Synthesis of 1.** To the solution of bis(4-(dimethylamino)phenyl)methanone (26.8 mg, 0.10 mmol) and malononitrile (19.8 mg, 0.30 mmol) in dichloromethane (10 mL) was added titanium tetrachloride (0.04 mL, 0.35 mmol) slowly at 0 $^o$C. After the reaction mixture was stirred for 30 min, pyridine (0.03 mL, 0.35 mmol) was injected and stirred for another 30 min. Then the mixture was heated at 40 $^o$C for 4 h. After the mixture was cooled down to room temperature, the reaction was quenched by water (10 mL) and the mixture was extracted with dichloromethane. The collected organic layer was washed with brine (20 mL), dried over $Na_2SO_4$ and concentrated under reduced pressure. The desired residue was purified by column chromatography using n-hexane/dichloromethane (10/1 to 1/1, v/v) as eluent to give the desired product **1** as a red solid (23.7 mg, 75.0% yield). $^1$H NMR (400 MHz, Chloroform-*d*) δ 7.40 (d, $J$ = 8.8 Hz, 1H), 6.69 (d, $J$ = 9.0 Hz, 1H), 3.10 (s, 3H). $^{13}$C NMR (101 MHz, $CDCl_3$) δ 173.92, 153.19, 133.57, 123.18, 117.24, 110.88, 69.45, 40.04. ESI-MS, m/z: $[M+Na]^+$ calcd 339.1586, found 339.1582 .

**Synthesis of 2.** To the solution of compound **3** (29.6 mg, 0.10 mmol) and malononitrile (19.8 mg, 0.30 mmol) in dichloromethane (10 mL) was added titanium tetrachloride (0.04 mL, 0.35 mmol) slowly at 0 $^{o}$C. After the reaction mixture was stirred for 30 min, pyridine (0.03 mL, 0.35 mmol) was injected and stirred for another 30 min. Then the mixture was heated at 40 $^{o}$C for 4 h. After the mixture was cooled down to room temperature, the reaction was quenched by water (10 mL) and the mixture was extracted with dichloromethane. The collected organic layer was washed with brine (20 mL), dried over $Na_2SO_4$ and concentrated under reduced pressure. The desired residue was purified by column chromatography using n-hexane/dichloromethane (8/1 to 1/1, v/v) as eluent to give the desired product **2** as a red solid (26.5 mg, 78.0% yield). $^{1}$H NMR (400 MHz, Chloroform-*d*) δ 7.77 – 7.21 (m, 4H), 6.70 – 5.77 (m, 4H), 4.00 – 3.30 (m, 8H), 2.40 – 1.99 (m, 4H).$^{13}$C NMR (101 MHz, $CDCl_3$) δ 194.09, 153.97, 150.91, 133.75, 133.41, 132.46, 132.06, 127.83, 126.88, 111.69, 111.30, 109.67, 109.50, 77.35, 77.03, 76.72, 51.79, 51.50, 42.43, 40.44, 31.73, 29.71, 22.70, 16.49, 14.13. ESI-MS, m/z: $[M+1]^+$ calcd 341.1766, found 341.1765

**Synthesis of 3.** A mixture of dibromobenzophenone (169.0 mg, 0.5 mmol), azetidine (85.0 mg, 1.5 mmol), caesium carbonate (1.14 g, 3.5 mmol), palladium(II) acetate (11.2 mg, 0.05 mmol), tri-tertbutylphosphine (30.3 mg, 0.15 mmol) and toluene (30 mL) was heated at 40$^{o}$C for 2 h. The reaction mixture was then heated at 110$^{o}$C for 24 h. After the mixture was cooled to room temperature, water (80 mL) and chloroform (200 mL) were added. The organic layer was separated and washed with brine, dried over anhydrous $MgSO_4$ and evaporated to dryness under reduced pressure. The crude product was purified by column chromatography on silica gel using hexane/toluene as eluent to afford **3** as a white solid in 60% yield (102 mg). $^{1}$H NMR (400 MHz, Chloroform-*d*) δ 7.63 (d, $J$ = 8.7 Hz, 4H), 6.30 (d, $J$ = 8.5 Hz, 4H), 3.89 (t, $J$ = 7.3 Hz, 8H), 2.43 – 2.22 (m, 4H). $^{13}$C NMR (101 MHz, $CDCl_3$) δ 194.20, 153.90, 132.02, 127.07, 109.49, 51.79, 16.69. ESI-MS, m/z: $[M+1]^+$ calcd 293.1648, found 293.1654.

## Machine Learning Methods

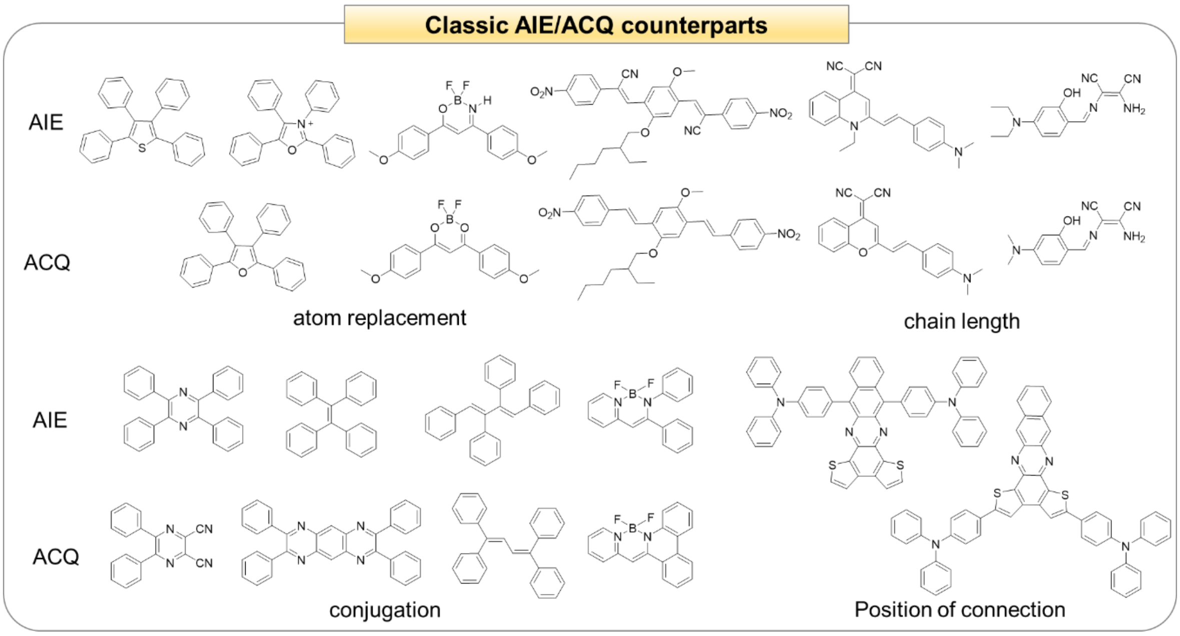


**Figure S1.** Examples of classic AIE/ACQ counterparts.

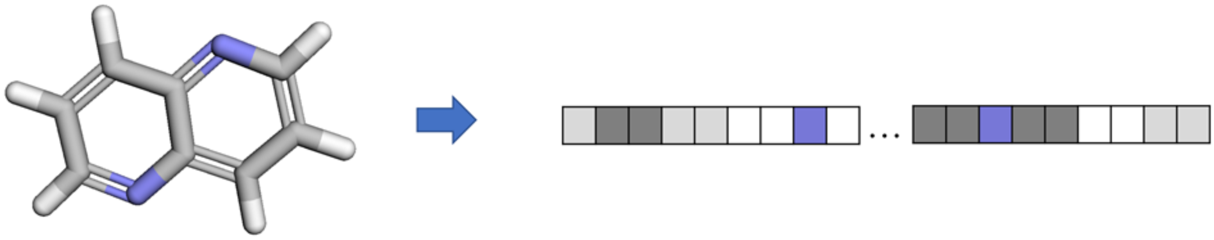

**Figure S2.** Encode a molecule into a series of binary digits. The SMILES of the molecule is "C1CNC2CCCNC2C1".

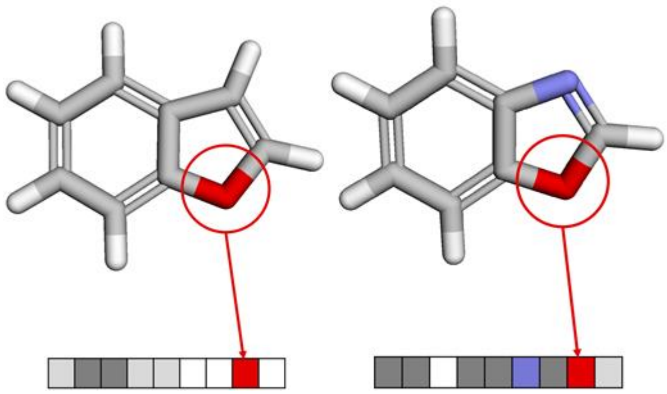

(a) C1CC2CCCCC2O1 (b) C1NC2CCCCC2O1

**Figure S3: The quantized molecules.**

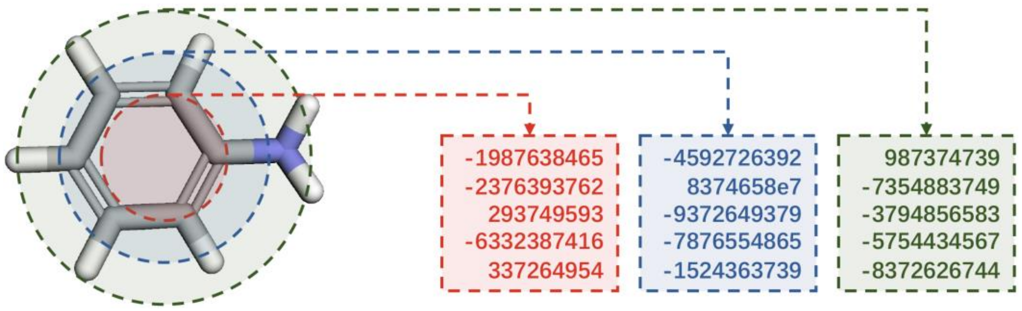


**Figure S4: Morgan circular fingerprint.**

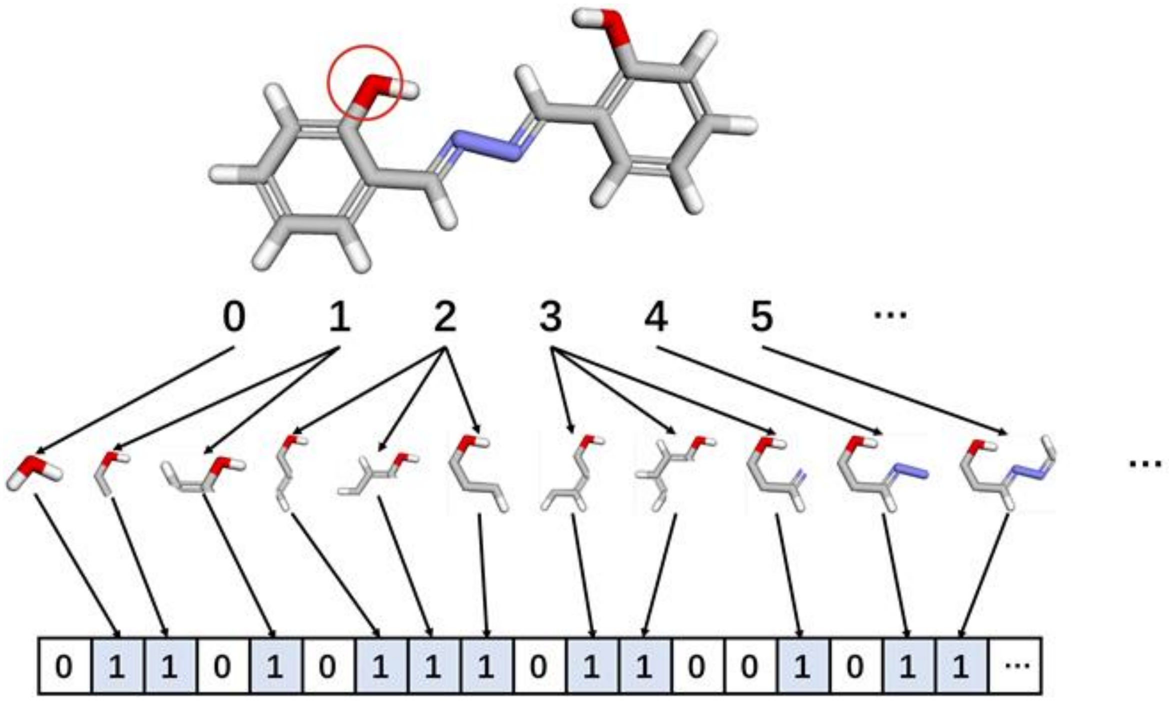


**Figure S5: Topological or path-based fingerprint.**

**Table S1: 1D and 2D quantitative descriptors.**

| NumTypeDescriptor | Description | Descriptor class |
|---|---|---|
| 1 1D FractionCSP3 | The fraction of C atoms that are SP3 hybridized. | Constitutional descriptor |

| | | |
|---|---|---|
| 2 1D HeavyAtomCount | Number of heavy atoms of a molecule. | Constitutional descriptor |
| 3 1D HeavyAtomMolWt | The average molecular weight of the molecule ignoring hydrogens | Constitutional descriptor |
| 4 1D NHOHCount | Number of NHs or OHs | Constitutional descriptor |
| 5 1D NOCount | Number of Nitrogens and Oxygens | Constitutional descriptor |
| 6 1D NumAliphaticCarbocycles | The number of aliphatic (containing at least one non-aromatic bond) carbocycles for a molecule | Constitutional descriptor |
| 7 1D NumAliphaticHeterocycles | The number of aliphatic (containing at least one non-aromatic bond) heterocycles for a molecule | Constitutional descriptor |
| 8 1D NumAliphaticRings | The number of aliphatic (containing at least one non-aromatic bond) rings for a molecule | Constitutional descriptor |
| 9 1D NumAromaticCarbocycles | The number of aromatic carbocycles for a molecule | Constitutional descriptor |
| 10 1D NumAromaticHeterocycles | The number of aromatic heterocycles for a molecule | Constitutional descriptor |
| 11 1D NumAromaticRings | The number of aromatic rings for a molecule | Constitutional descriptor |
| 12 1D NumHAcceptors | Number of Hydrogen Bond Acceptors | Constitutional descriptor |
| 13 1D NumHDonors | Number of Hydrogen Bond Donors | Constitutional descriptor |
| 14 1D NumHeteroatoms | Number of Heteroatoms | Constitutional descriptor |
| 15 1D NumRadicalElectrons | The number of radical electrons the molecule has (says nothing about spin state) | Constitutional descriptor |
| 16 1D NumRotatableBonds | Number of Rotatable Bonds | Constitutional descriptor |
| 17 1D NumSaturatedCarbocycles | The number of saturated carbocycles for a molecule | Constitutional descriptor |
| 18 1D NumSaturatedHeterocycles | The number of saturated heterocycles for a molecule | Constitutional descriptor |
| 19 1D NumSaturatedRings | The number of saturated rings for a molecule | Constitutional descriptor |
| 20 1D NumValenceElectrons | The number of valence electrons the molecule has | Constitutional descriptor |
| 21 1D RingCount | The number of rings for a molecule | Constitutional descriptor |
| 22 2D BalabanJ | Balaban's J value for a molecule.[14] | Topological descriptor |
| 23 2D BertzCT | A topological index meant to quantify "complexity" of molecules. [15] | Topological descriptor |
| 24 2D Chi0 | From equations (1),(9) and (10) of. [16] | Connectivity descriptor |
| 25 2D Chi0n | Similar to Hall Kier Chi0v, but uses nVal instead of valence. [16] | Connectivity descriptor |
| 26 2D Chi0v | From equations (5),(9) and (10) of. [16] | Connectivity descriptor |
| 27 2D Chi1 | From equations (1),(11) and (12) of. [16] | Connectivity descriptor |
| 28 2D Chi1n | Similar to Hall Kier Chi1v, but uses nVal instead of valence. [16] | Connectivity descriptor |
| 29 2D Chi1v | From equations (5),(11) and (12) of. [16] | Connectivity descriptor |
| 30 2D Chi2n | Similar to Hall Kier Chi2v, but uses nVal instead of valence. [16] | Connectivity descriptor |
| 31 2D Chi2v | From equations (5),(15) and (16) of. [16] | Connectivity descriptor |
| 32 2D Chi3n | Similar to Hall Kier Chi3v, but uses nVal instead of valence. [16] | Connectivity descriptor |
| 33 2D Chi3v | From equations (5),(15) and (16) of. [16] | Connectivity descriptor |

| | | |
|---|---|---|
| 34 2D Chi4n | Similar to Hall Kier Chi4v, but uses nVal instead of valence. [16] | Connectivity descriptor |
| 35 2D Chi4v | From equations (5),(15) and (16) of [16] | Connectivity descriptor |
| 36 2D EState_VSA10 | MOE-type descriptors using EState indices and surface area contributions. | MOE-type descriptor |
| 37 2D EState_VSA11 | MOE-type descriptors using EState indices and surface area contributions. | MOE-type descriptor |
| 38 2D EState_VSA1 | MOE-type descriptors using EState indices and surface area contributions. | MOE-type descriptor |
| 39 2D EState_VSA2 | MOE-type descriptors using EState indices and surface area contributions. | MOE-type descriptor |
| 40 2D EState_VSA3 | MOE-type descriptors using EState indices and surface area contributions. | MOE-type descriptor |
| 41 2D EState_VSA4 | MOE-type descriptors using EState indices and surface area contributions. | MOE-type descriptor |
| 42 2D EState_VSA5 | MOE-type descriptors using EState indices and surface area contributions. | MOE-type descriptor |
| 43 2D EState_VSA6 | MOE-type descriptors using EState indices and surface area contributions. | MOE-type descriptor |
| 44 2D EState_VSA7 | MOE-type descriptors using EState indices and surface area contributions. | MOE-type descriptor |
| 45 2D EState_VSA8 | MOE-type descriptors using EState indices and surface area contributions. | MOE-type descriptor |
| 46 2D EState_VSA9 | MOE-type descriptors using EState indices and surface area contributions. | MOE-type descriptor |
| 47 2D ExactMolWt | The molecule's exact molecular weight. | Molecular property descriptor |
| 48 2D FpDensityMorgan1 | Morgan fingerprint density | Topological descriptor |
| 49 2D FpDensityMorgan2 | Morgan fingerprint density | Topological descriptor |
| 50 2D FpDensityMorgan3 | Morgan fingerprint density | Topological descriptor |
| 51 2D HallKierAlpha | The Hall-Kier alpha value for a molecule. [16] | Topological descriptor |
| 52 2D Ipc | The information of characteristic polynomial coefficients in the adjacency matrix of molecular hydrogen graphs. | Topological descriptor |
| 53 2D Kappa1 | Hall-Kier Kappa1 value | Topological descriptor |
| 54 2D Kappa2 | Hall-Kier Kappa2 value | Topological descriptor |
| 55 2D Kappa3 | Hall-Kier Kappa3 value | Topological descriptor |
| 56 2D LabuteASA | Labute's Approximate Surface Area (ASA from MOE) | MOE-type descriptor |
| 57 2D MaxEStateIndex | Returns a tuple of EState indices for the molecule. [17] | Topological descriptor |
| 58 2D MinEStateIndex | Returns a tuple of EState indices for the molecule. [17] | Topological descriptor |

| | | |
|---|---|---|
| 59 2D MolLogP | Wildman-Crippen LogP value. [18] | Molecular property descriptor |
| 60 2D MolMR | Wildman-Crippen MR value. [18] | Molecular property descriptor |
| 61 2D MolWt | The average molecular weight of the molecule | Molecular property descriptor |
| 61 2D PEOE_VSA1 | MOE Charge VSA Descriptor 1 | MOE-type descriptor |
| 63 2D PEOE_VSA10 | MOE Charge VSA Descriptor 10 | MOE-type descriptor |
| 64 2D PEOE_VSA11 | MOE Charge VSA Descriptor 11 | MOE-type descriptor |
| 65 2D PEOE_VSA12 | MOE Charge VSA Descriptor 12 | MOE-type descriptor |
| 66 2D PEOE_VSA13 | MOE Charge VSA Descriptor 13 | MOE-type descriptor |
| 67 2D PEOE_VSA14 | MOE Charge VSA Descriptor 14 | MOE-type descriptor |
| 68 2D PEOE_VSA2 | MOE Charge VSA Descriptor 2 | MOE-type descriptor |
| 69 2D PEOE_VSA3 | MOE Charge VSA Descriptor 3 | MOE-type descriptor |
| 70 2D PEOE_VSA4 | MOE Charge VSA Descriptor 4 | MOE-type descriptor |
| 71 2D PEOE_VSA5 | MOE Charge VSA Descriptor 5 | MOE-type descriptor |
| 72 2D PEOE_VSA6 | MOE Charge VSA Descriptor 6 | MOE-type descriptor |
| 73 2D PEOE_VSA7 | MOE Charge VSA Descriptor 7 | MOE-type descriptor |
| 74 2D PEOE_VSA8 | MOE Charge VSA Descriptor 8 | MOE-type descriptor |
| 75 2D PEOE_VSA9 | MOE Charge VSA Descriptor 9 | MOE-type descriptor |
| 76 2D qed | Quantitative estimation of drug-likeness | Topological descriptor |
| 77 2D SlogP_VSA1 | MOE logP VSA Descriptor 1 | MOE-type descriptor |
| 78 2D SlogP_VSA10 | MOE logP VSA Descriptor 10 | MOE-type descriptor |
| 79 2D SlogP_VSA11 | MOE logP VSA Descriptor 11 | MOE-type descriptor |
| 80 2D SlogP_VSA12 | MOE logP VSA Descriptor 12 | MOE-type descriptor |
| 81 2D SlogP_VSA2 | MOE logP VSA Descriptor 2 | MOE-type descriptor |
| 82 2D SlogP_VSA3 | MOE logP VSA Descriptor 3 | MOE-type descriptor |
| 83 2D SlogP_VSA4 | MOE logP VSA Descriptor 4 | MOE-type descriptor |
| 84 2D SlogP_VSA5 | MOE logP VSA Descriptor 5 | MOE-type descriptor |
| 85 2D SlogP_VSA6 | MOE logP VSA Descriptor 6 | MOE-type descriptor |
| 86 2D SlogP_VSA7 | MOE logP VSA Descriptor 7 | MOE-type descriptor |
| 87 2D SlogP_VSA8 | MOE logP VSA Descriptor 8 | MOE-type descriptor |
| 88 2D SlogP_VSA9 | MOE logP VSA Descriptor 9 | MOE-type descriptor |
| 89 2D SMR_VSA10 | MOE MR VSA Descriptor 10 | MOE-type descriptor |
| 90 2D SMR_VSA1 | MOE MR VSA Descriptor 1 | MOE-type descriptor |
| 91 2D SMR_VSA2 | MOE MR VSA Descriptor 2 | MOE-type descriptor |
| 92 2D SMR_VSA3 | MOE MR VSA Descriptor 3 | MOE-type descriptor |
| 93 2D SMR_VSA4 | MOE MR VSA Descriptor 4 | MOE-type descriptor |

| | | |
|---|---|---|
| 94 2D SMR_VSA5 | MOE MR VSA Descriptor 5 | MOE-type descriptor |
| 95 2D SMR_VSA6 | MOE MR VSA Descriptor 6 | MOE-type descriptor |
| 96 2D SMR_VSA7 | MOE MR VSA Descriptor 7 | MOE-type descriptor |
| 97 2D SMR_VSA8 | MOE MR VSA Descriptor 8 | MOE-type descriptor |
| 98 2D SMR_VSA9 | MOE MR VSA Descriptor 9 | MOE-type descriptor |
| 99 2D TPSA | Topological polar surface area | Molecular property descriptor |
| 100 2D VSA_EState1 | VSA EState Descriptor 1 | MOE-type descriptor |
| 101 2D VSA_EState10 | VSA EState Descriptor 10 | MOE-type descriptor |
| 102 2D VSA_Estate2 | VSA EState Descriptor 2 | MOE-type descriptor |
| 103 2D VSA_Estate3 | VSA EState Descriptor 3 | MOE-type descriptor |
| 104 2D VSA_Estate4 | VSA EState Descriptor 4 | MOE-type descriptor |
| 105 2D VSA_Estate5 | VSA EState Descriptor 5 | MOE-type descriptor |
| 106 2D VSA_Estate6 | VSA EState Descriptor 6 | MOE-type descriptor |
| 107 2D VSA_Estate7 | VSA EState Descriptor 7 | MOE-type descriptor |
| 108 2D VSA_Estate8 | VSA EState Descriptor 8 | MOE-type descriptor |
| 109 2D VSA_Estate9 | VSA EState Descriptor 9 | MOE-type descriptor |

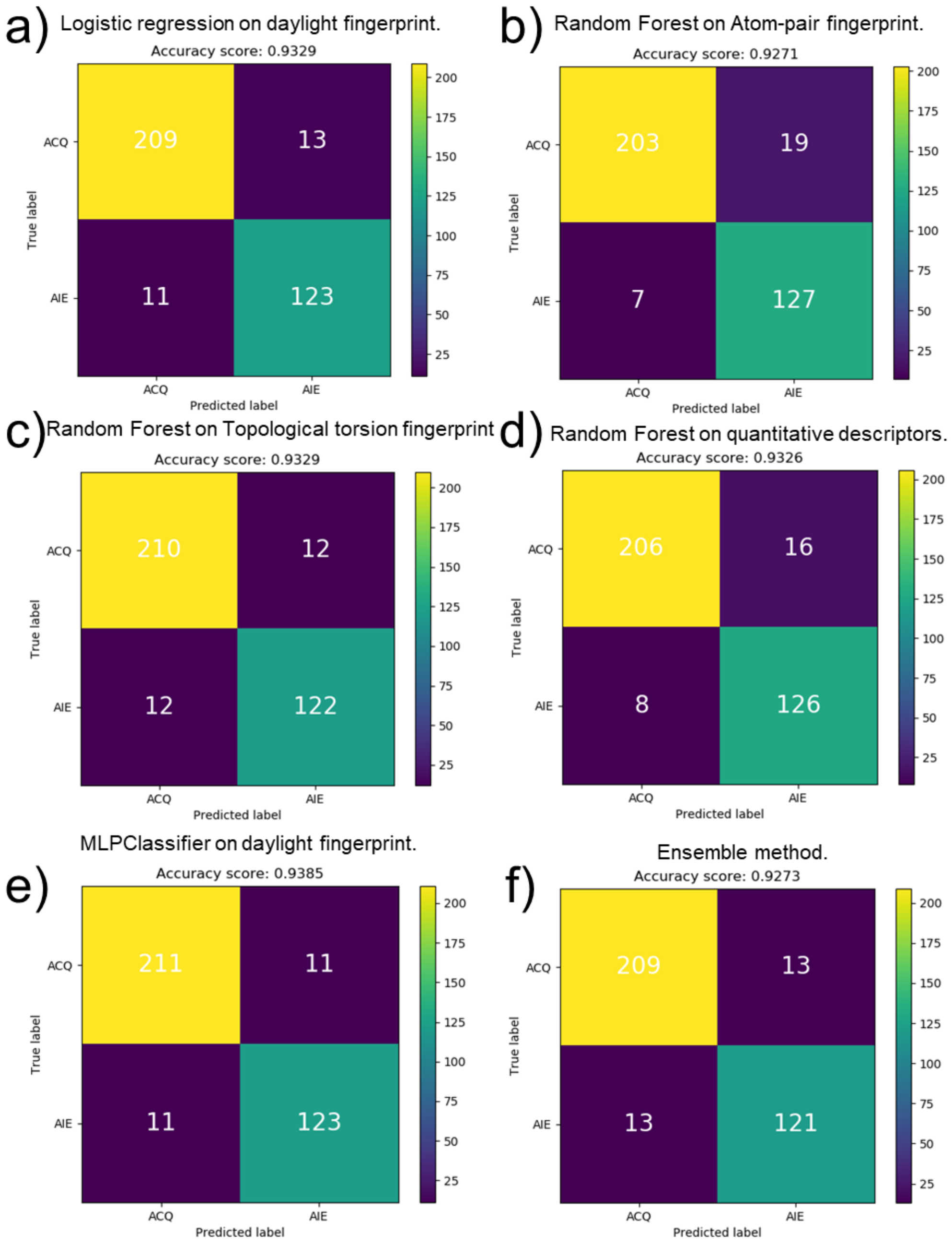


**Figure S6**. Confounding matrix of Ensemble method and competing methods based on single-modal descriptors.

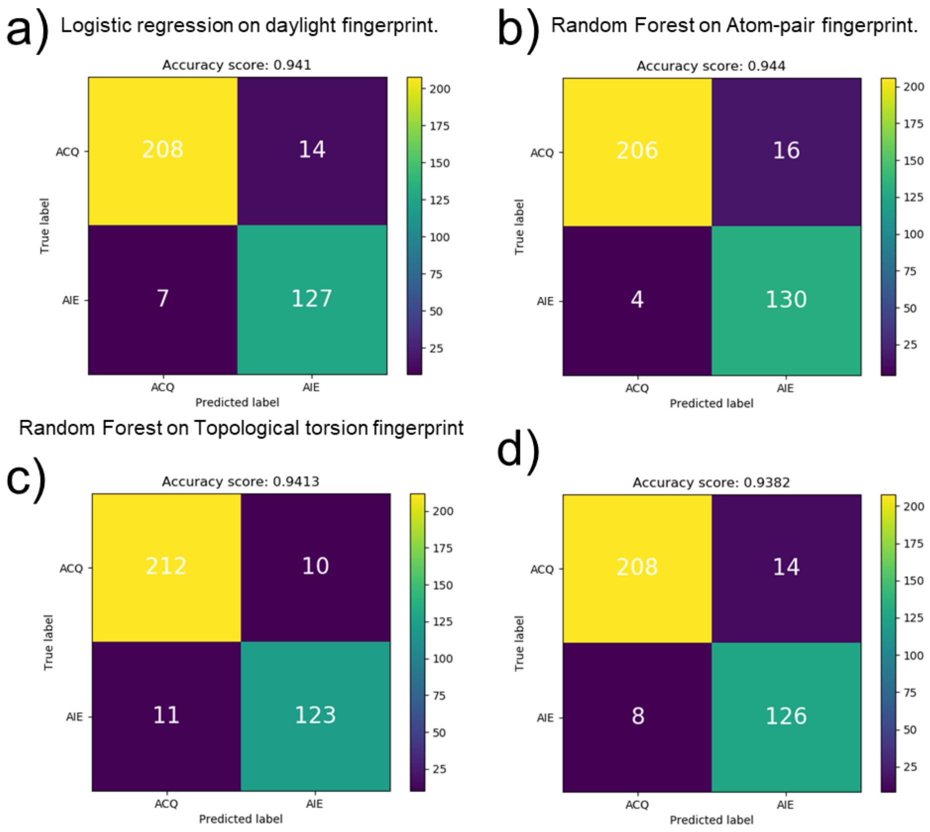


**Figure S7.** Confounding matrix of Ensemble method and competing methods based on multi-modal descriptors.

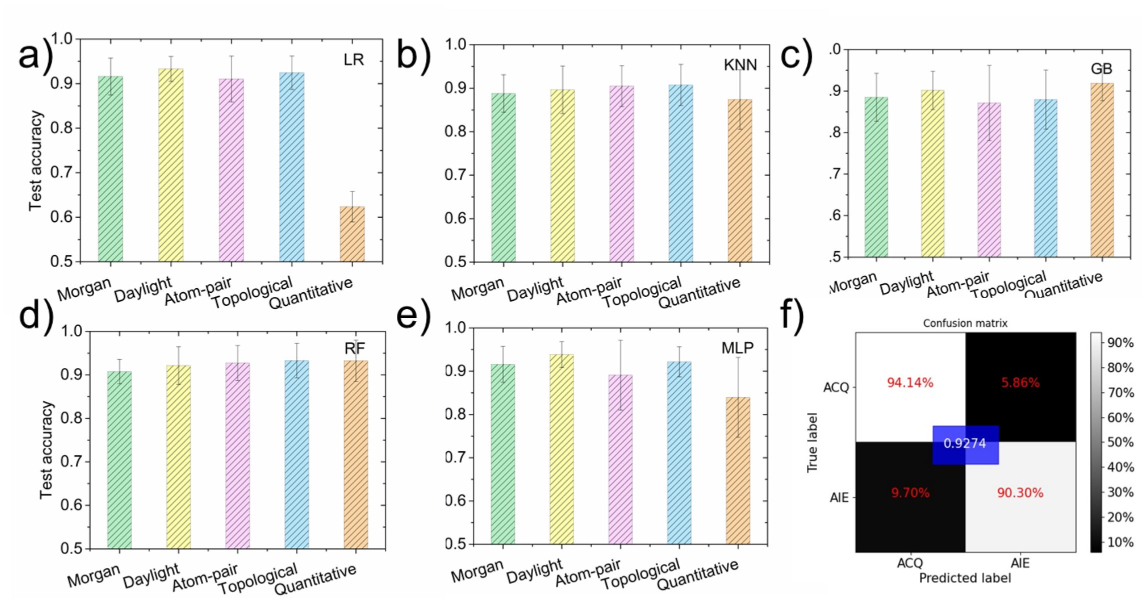

**Figure S8**. (a-e) Test accuracy of five methods with different descriptors. (f) Confounding matrix of Ensemble method base on single-modal descriptor.

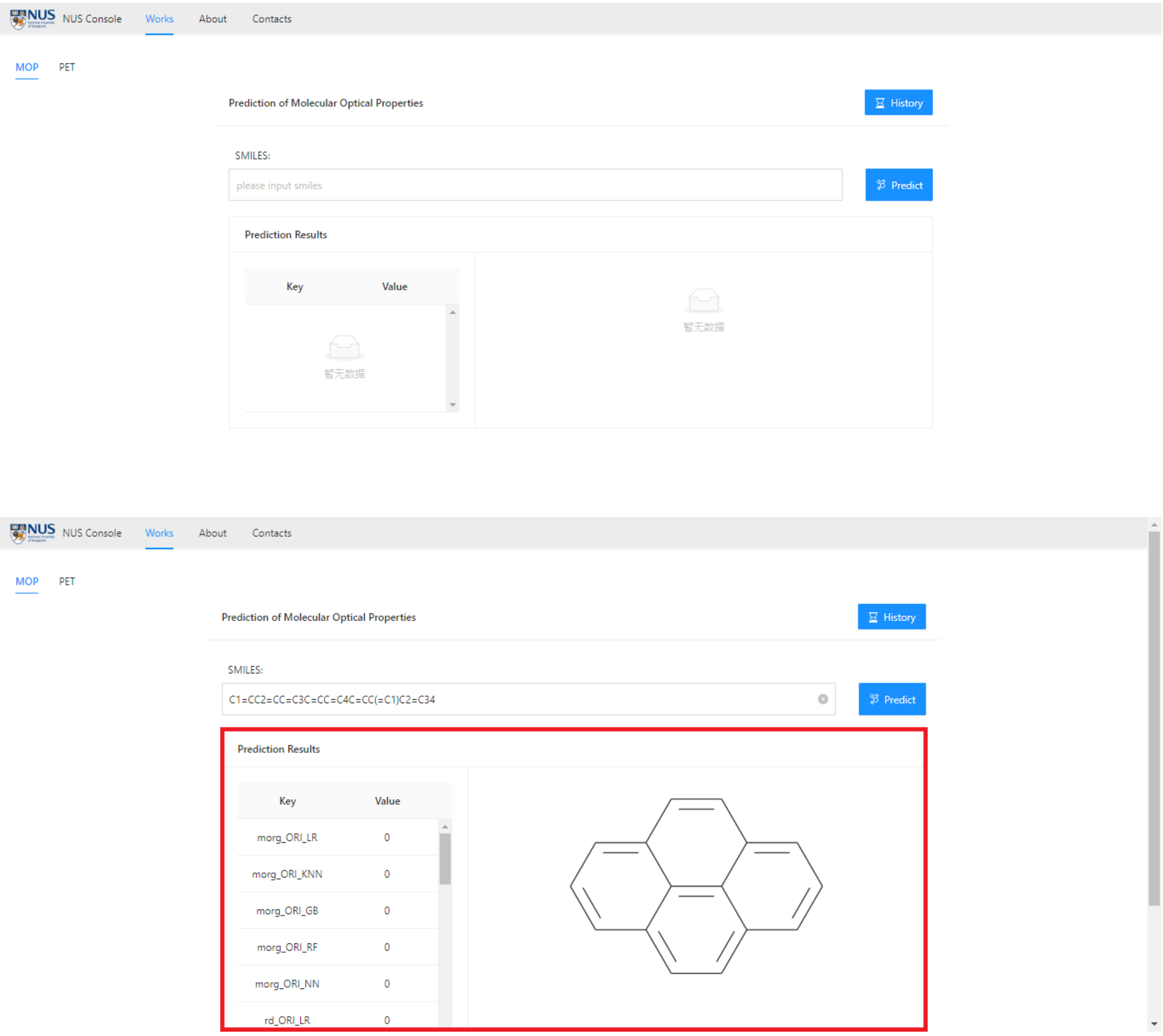


**Figure S9**. UI of the prediction system.1 and 0 represent AIE and ACQ, respectively. (https://aiapp.gaha.xyz:7443/nus/html/#/works)

**Benchmark against SELF-referencIng Embedded Strings**

Krenn, Mario, et al. [S1] introduced a string-based representation of molecular, SELFIES (SELF-referencIng Embedded Strings). The motivation for proposing SELFIES is that current generative models are hard to generate valid SMILES molecules, even if sufficient diversity

of molecular SMILES data is trained. The reason for this problem is that SMILES describes not only the string representation of the molecules, but also information between different components, such as bonds and rings. SELFIES is robust precisely because it considers only the embedding of strings, ignoring the correlations between the different components in the molecule. However, SELFIES does not have such a generalization property as SMILES. Besides, SELFIES is only quantitatively evaluated based on the results of the algorithms. The validity of SELFIES needs to be further tested in chemical laboratory experiments.

In ref [S3], Nigam, A. et al. proposed the STONED algorithm, a simple algorithm to perform interpolation and exploration in the chemical space. The author implemented STONED and other comparable methods based on SELFIES molecular representation, making it easier to obtain the valid representation of string embedding. Experiments confirmed that the combination of STONED and SELFIES could produce considerable results. However, what STONED achieved was rediscovery, which is to modify the original molecular expression. Specifically, this means modifying an existing molecule by interpolation, modifying only parts of the molecule, without reconstructing a new molecule from characters or fragments. In this way, the model can only generate molecules with modified parts of their composition, without the ability to learn and create molecules.

Chem-VAE was proposed in ref [S1] and is widely used as a baseline comparison method in molecular generation work. Ref [S2] and ref [S3] use Chem-VAE to verify the effectiveness of SELFIES and STONED. Chem-VAE simply applies VAE to molecular research through one-hot encoding. First, the vector was encoded into a high-dimensional vector, then the potential vector is obtained through the normalization of transformation, and finally the vector is decoded back to the molecule using decoding blocks. The author emphasizes that the current challenge is how to generate valid molecules, and VAE is expected to achieve this.

However, the results of Chem-VAE were not ideal, probably because it did not take into account the connection and sequence of the molecules. For the molecules generated using the variational autoencoder in the experiment, the latent space points were encoded by 1000 seed molecules, which are from ZINC data. This means that a code is generated from a valid molecule to a latent vector, and then the vector is decoded to a new molecule. From the algorithm evaluation perspective, if the molecule is decoded back to the original molecule, it proves that the model learns well and can accurately decode the molecule back to the input molecule. If there were different parts from the input molecules, that is, the mutation parts, it means that the algorithm is creative and the results can be diversified. Therefore, it is not easy to evaluate the VAE model quantitatively.

Actually, we have done a lot of research in this area and read a lot of literature, such as [S4-S7]. In these methods, the target tasks are generated from random latent vectors, randomly generating a latent vector with Gaussian distribution, then decoding this vector to the target. We implemented Chem-VAE (the author published the code[S8]), transformer-VAE (code by ourselves, method architecture in ref [S6]), and the method proposed by ourselves on ZINC. We generated 1000 samples from random latent vectors for all the methods. The results are listed as follows:

**Chem-VAE**

None of the 1000 samples generated is a valid SMILES.

An example:

The dimension of latent vector is 1 × 292:

[0.0043497234582901, -0.015666436403989792, 0.004856988321989775, …]

The corresponding molecule is:

*CCB)lFBBIS]C##CC8#I]]###B\lBB4CCB4CC8BI]-8B\CC*

**Transformer-VAE**

None of the 1000 samples generated is a valid SMILES.

An example:

The dimension of latent vector is 16 × 16:

[[0.30958104133605957, 1.0263967514038086, 0.9525277614593506, …]]

The corresponding molecule is:

*(-PS(Nc(CC(-\]\N(cc3ccON@=-3)N(c(Cc2-O[Nc2PSc2c(-cc(2SN*

**Our proposed sequence model**

952 valid SMILES are achieved. Here, we listed two valid examples:

The dimension of latent vector is 1 × 32:

[-0.6104545593261719, 1.0966098308563232, 0.2640085816383362, …]

The corresponding molecules are:

*C[NH2+][C@@]1(C(=O)[O-])CCC[C@@H]([NH+]2CC[C@@H](C)[C@H](SC)C2)C1*

*CNc1nc(NC(=O)c2nccnc2N)nc(C)c1Br*

These are shown in Figure S10.

It can be seen from the experimental results that it is feasible to generate effective SMILES. The key is to find a suitable algorithm to learn the dataset efficiently. For our AIE dataset, we also applied Chem-VAE and proposed our method to conduct experiments. The results are as follows:

**Chem-VAE**

None of the 1000 samples generated is a valid SMILES.

An example:

The dimension of latent vector is 1 × 292:

[-0.0005187964416109025, -0.008428120985627174, 0.022790968418121338, …]

The corresponding molecule is:

*9 :: 99999999999999999999999:::*

**Our proposed fragment method**

All the generated molecules are valid. Here, we listed two valid examples:

The dimension of latent vector is 20 × 24:

[-1.5255959033966064, -0.7502318024635315, -0.6539809107780457, …]

The corresponding molecules are:

*CCN1C(C=Cc2ccc(-c3ccc(N(c4ccccc4)c4ccccc4)cc3)s2)=CC(=C(C#N)C#N)c2ccccc*

*21*

*CCn1c2ccccc2c2cc(C=C3C(=O)c4ccccc4C3=O)ccc21*

These are shown in Figure S11.

In this experiment, we also used 356 samples for training. The results clearly indicate that the Chem-VAE algorithm cannot be optimized on small datasets, which the author also pointed out in the paper. But our proposed fragment method can work very well, which further illustrates that the number of our datasets is sufficient to train the model, including both prediction and generation. The information in SMILES can be used for in-depth study of molecular properties and we think this is a direction that the community should pay more attention in further research.

The above is the discussion of the problem of molecule generation, that is, the inverse question. Now we want to summarize the roles played by forward prediction and inverse generation. When a generative model is trained, it is easy to generate molecules. Ten of

thousands of molecules can be generated with only one line of code and simple operations. Just like the model we trained on the AIE dataset. But it is hard to verify and obtain the required molecules. For example, after we generate molecules, they do not make sense if we do not verify and label them. We have used our prediction model proposed in this paper as a discriminator to predict our generated molecules, and then every molecule have its label, AIE/ACQ. This completes the process of the whole flowchart for molecular generation in this field.

In addition to generation, we also proposed a discovery strategy. We further added elements that do not exist in the current molecular dataset to be reasonably combined into unseen molecules. Here, we just want to show that it is impossible to rely solely on chemists to do experimental verification, but prediction and learning can be used for analysis. Therefore, the forward algorithm can be used to preliminary screen the generated molecules, provide a preliminary analysis of their properties, and further manual analysis. In ref [S1], the authors also trained a model for predicting molecular properties to measure molecules. In short, only by achieving good results in two directions at the same time, we can truly implement end-to-end algorithms to simulate human thoughts. Therefore, research in these two directions is equally essential, and the key is how to apply the model to real data. Our work is based on real data and problems. In future work, our goal is to improve the closed-loop analysis of molecules, so that the model has the ability of generation and analysis and prediction at the same time.

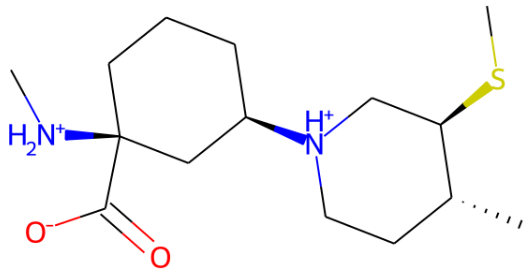


(a)

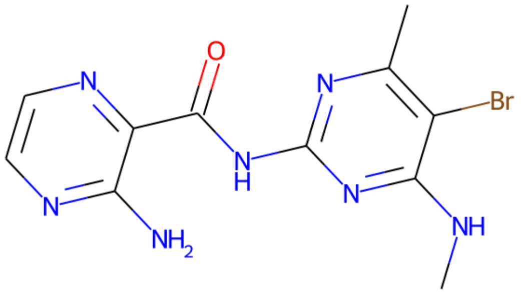


(b)

**Figure S10.** Plots of molecules generated by our proposed method on ZINC dataset

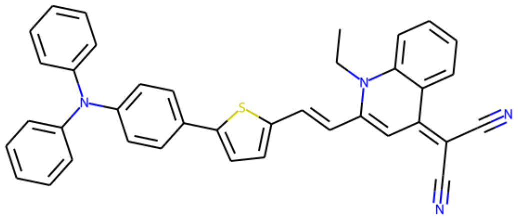


(a)

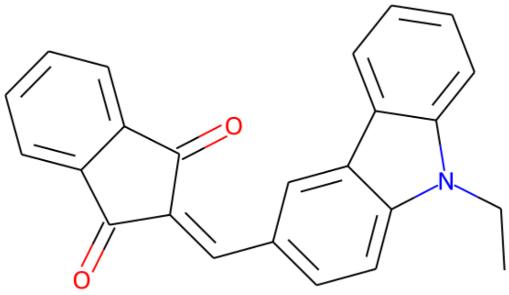


(b)

**Figure S11**. Plots of molecules generated by our proposed method on AIE dataset

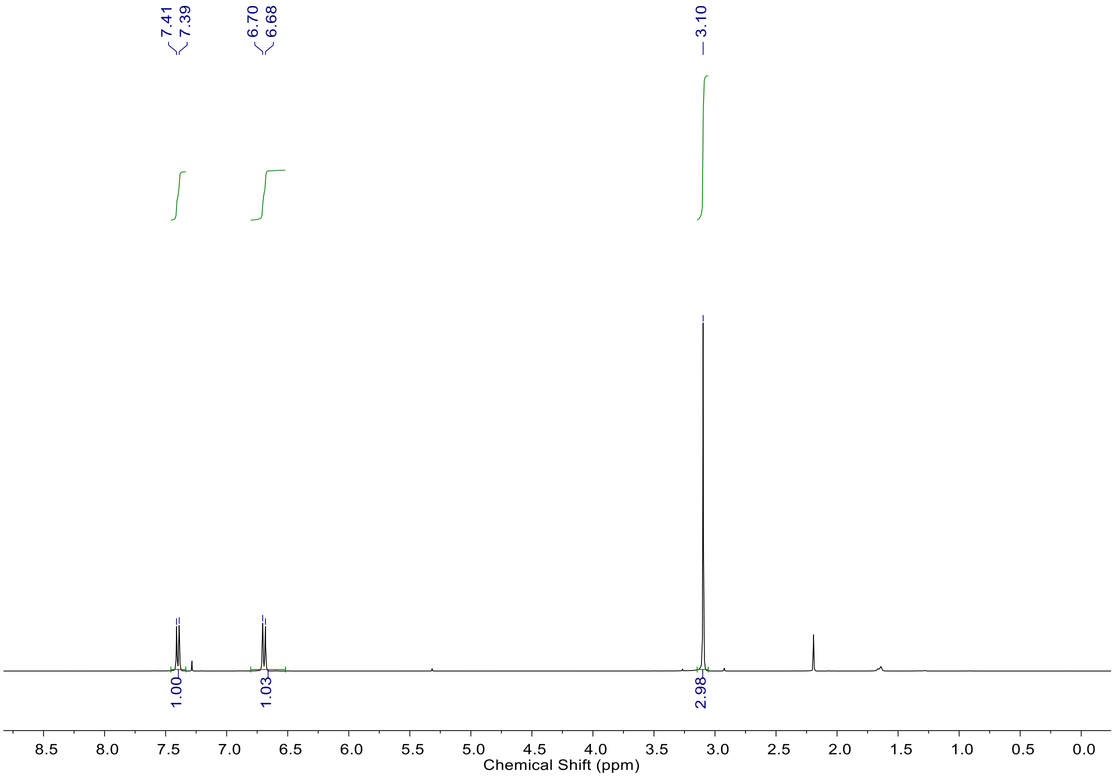


**Figure S12**. The $^{1}H$ NMR spectrum of **1** in in $CDCl_3$.

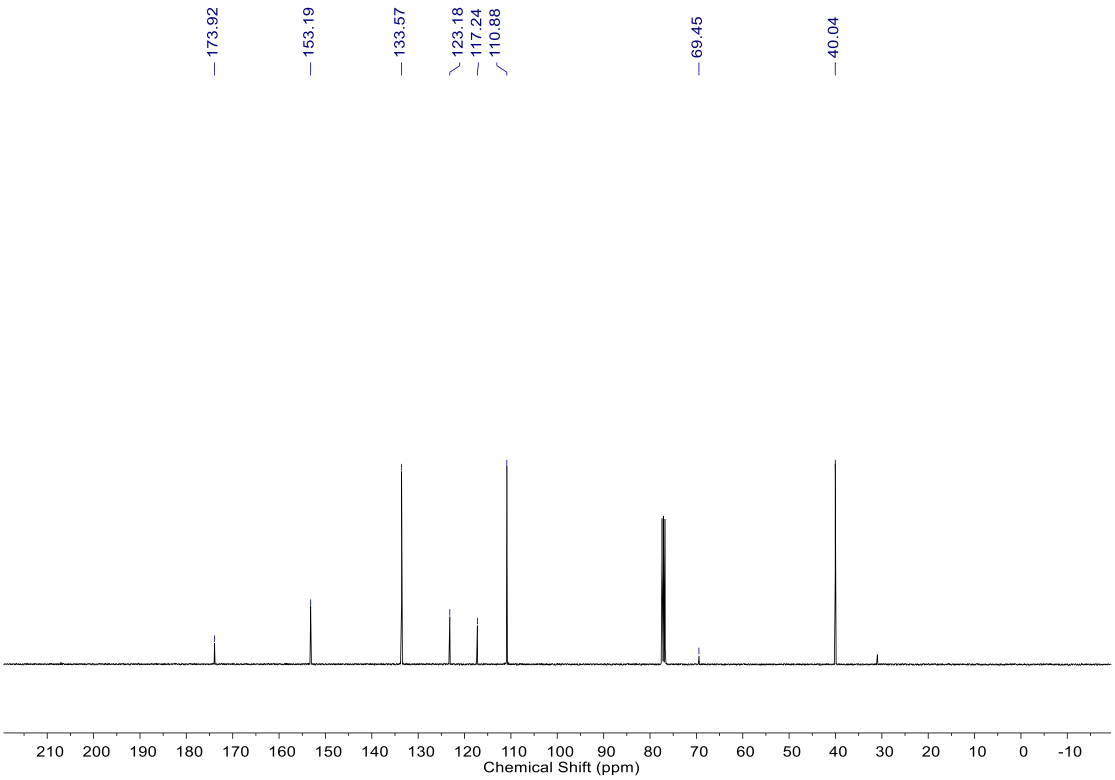


**Figure S13**. The $^{13}C$ NMR spectrum of **1** in $CDCl_3$.

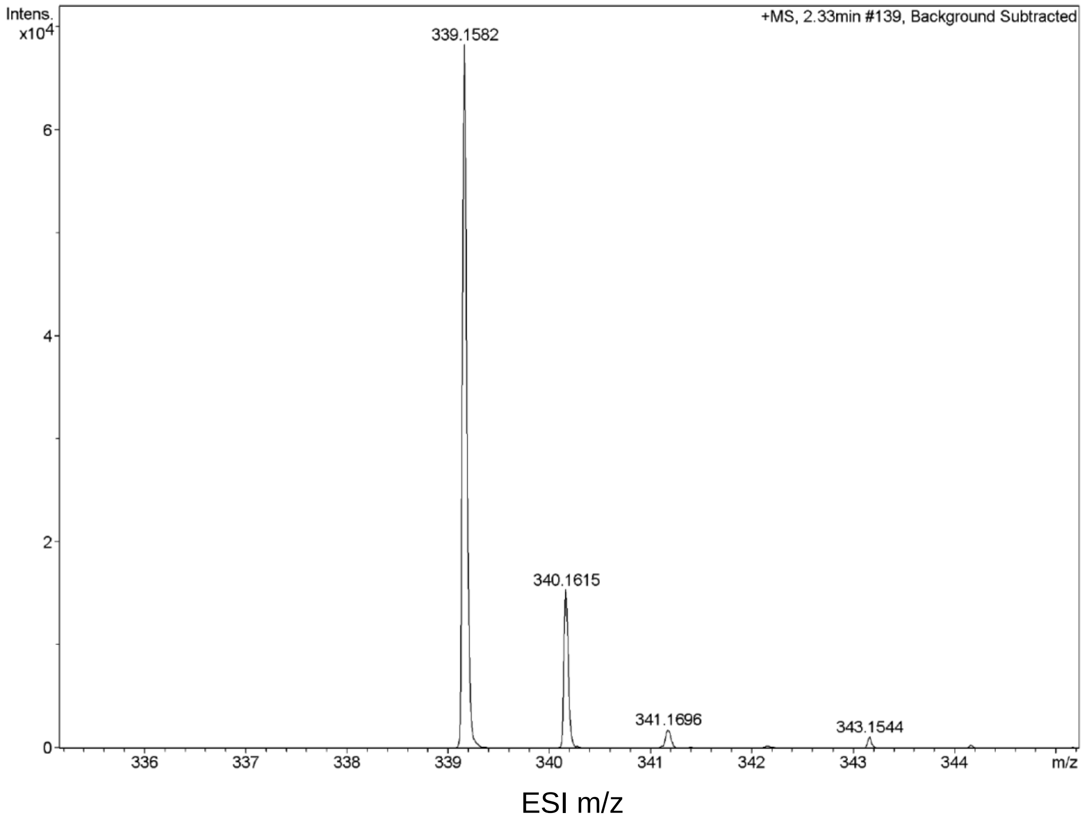


**Figure S14**. The HRMS of **1**.

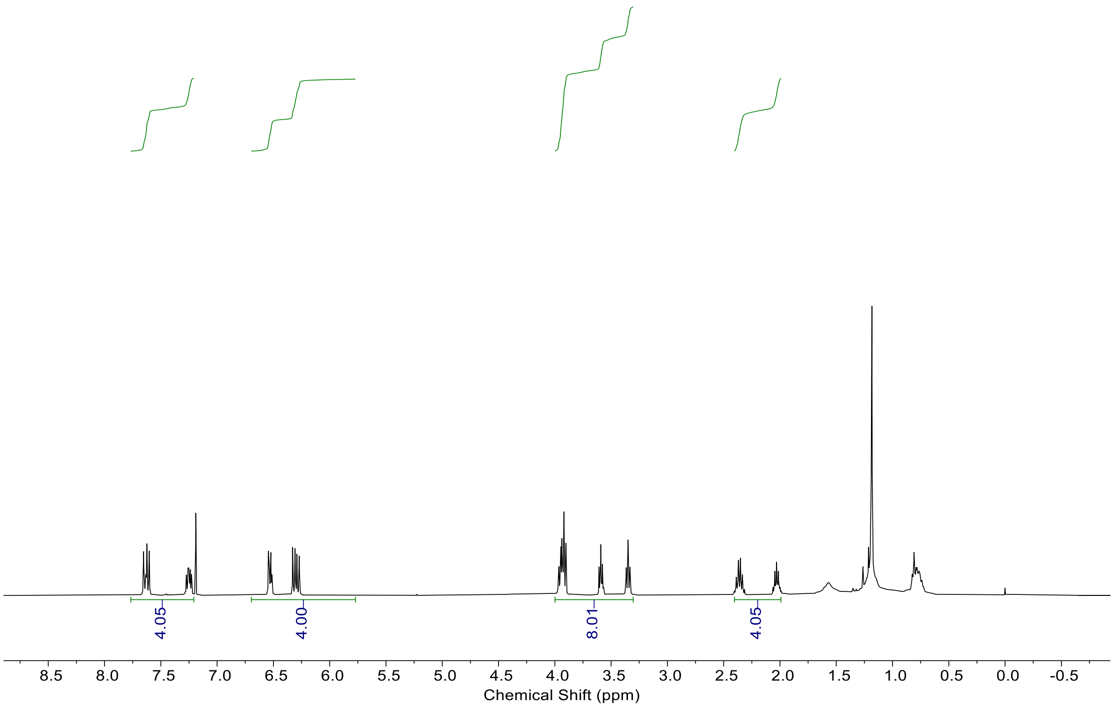


**Figure S15**. The $^{1}H$ NMR spectrum of **2** in in $CDCl_3$.

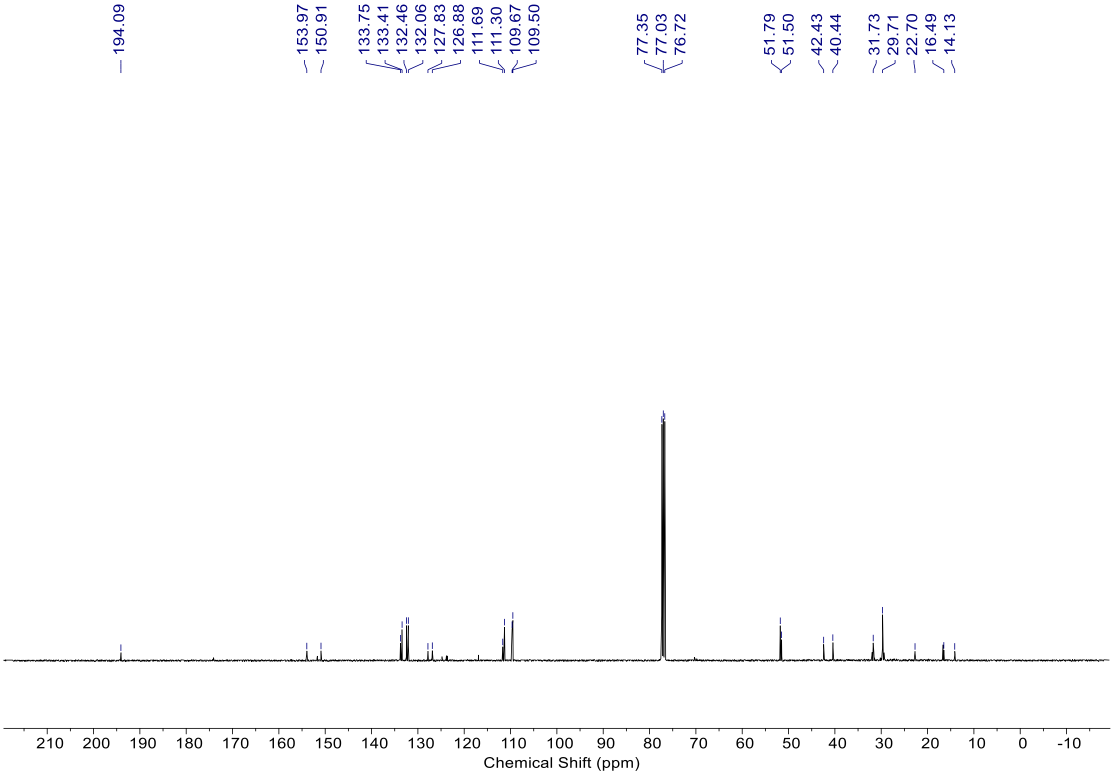


**Figure S16**. The $^{13}$C NMR spectrum of **2** in $CDCl_3$.

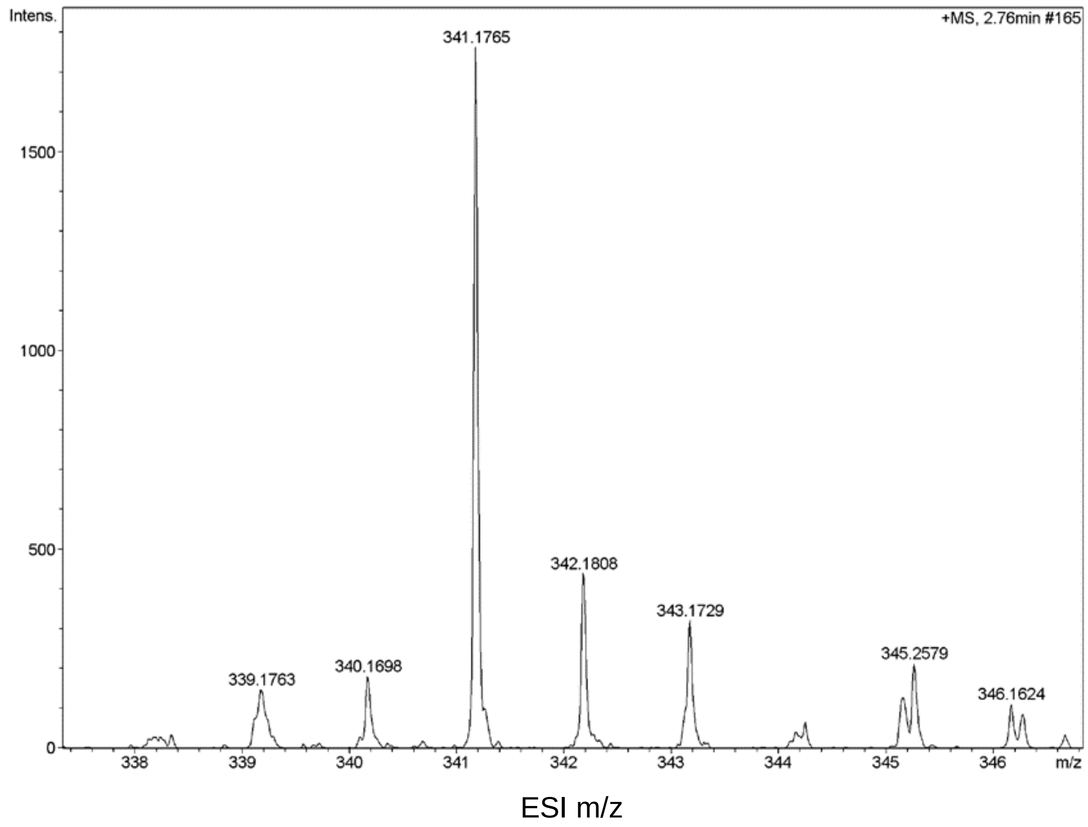


**Figure S17**. The HRMS of **2**.

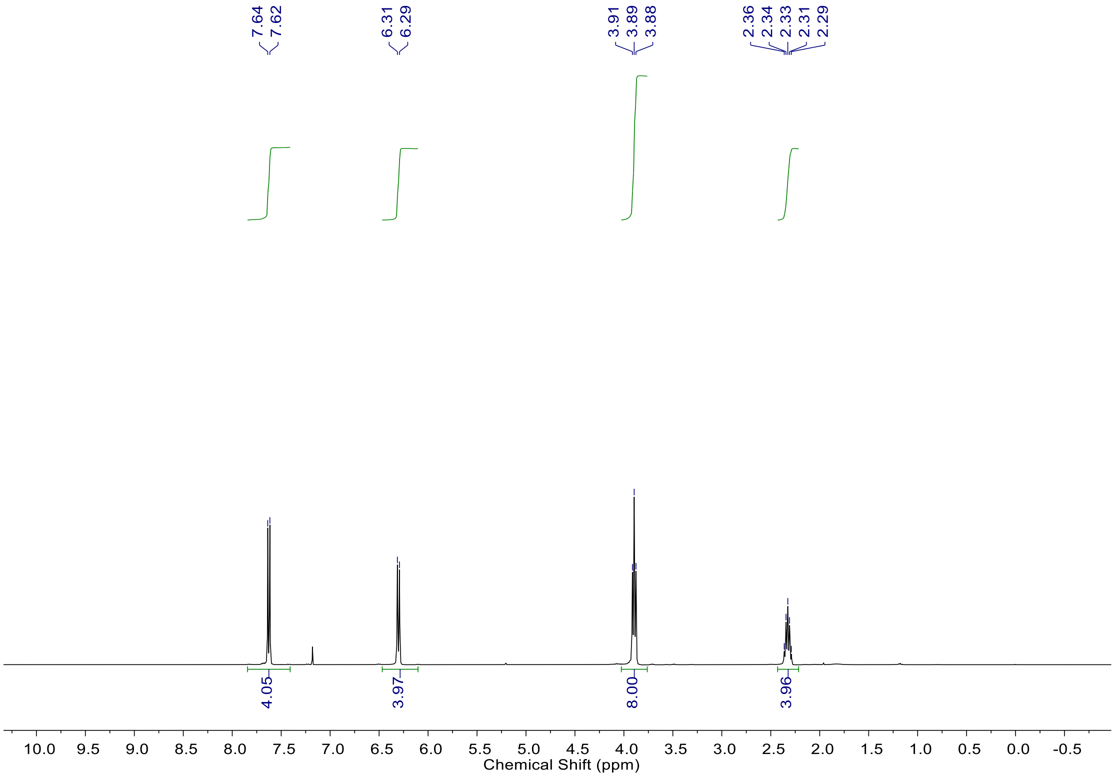


**Figure S18**. The $^{1}$H NMR spectrum of **3** in in $CDCl_3$.

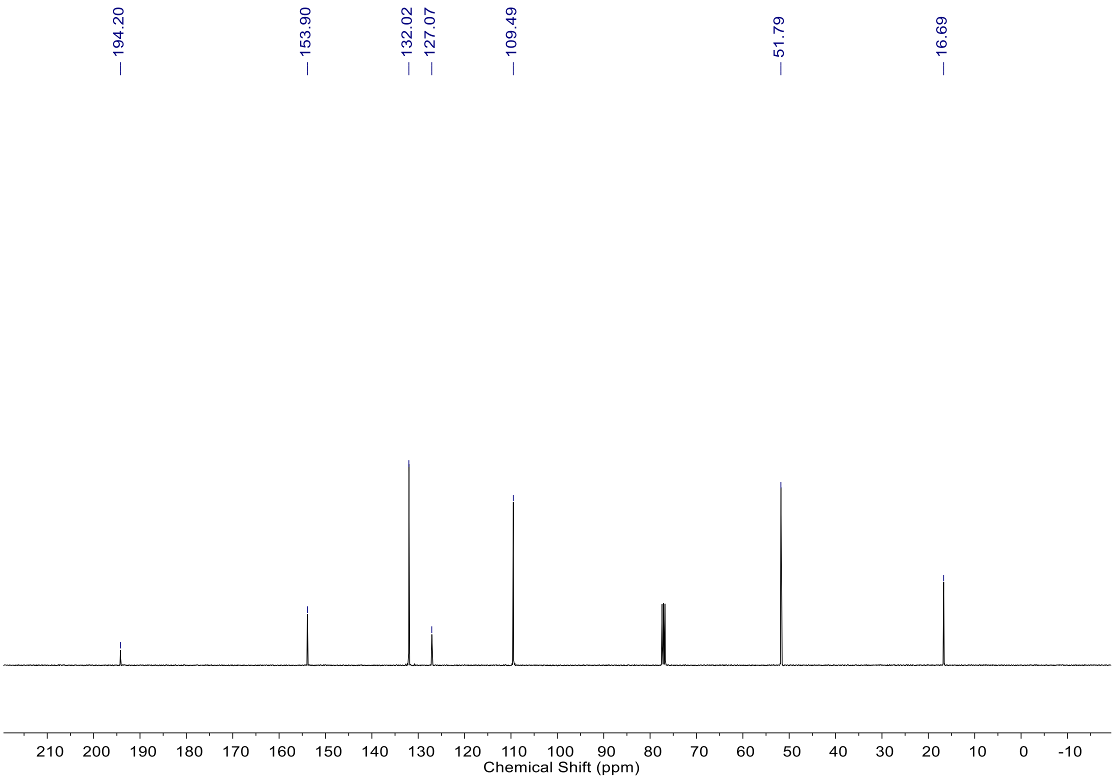


**Figure S19**. The $^{13}$C NMR spectrum of **3** in $CDCl_3$.

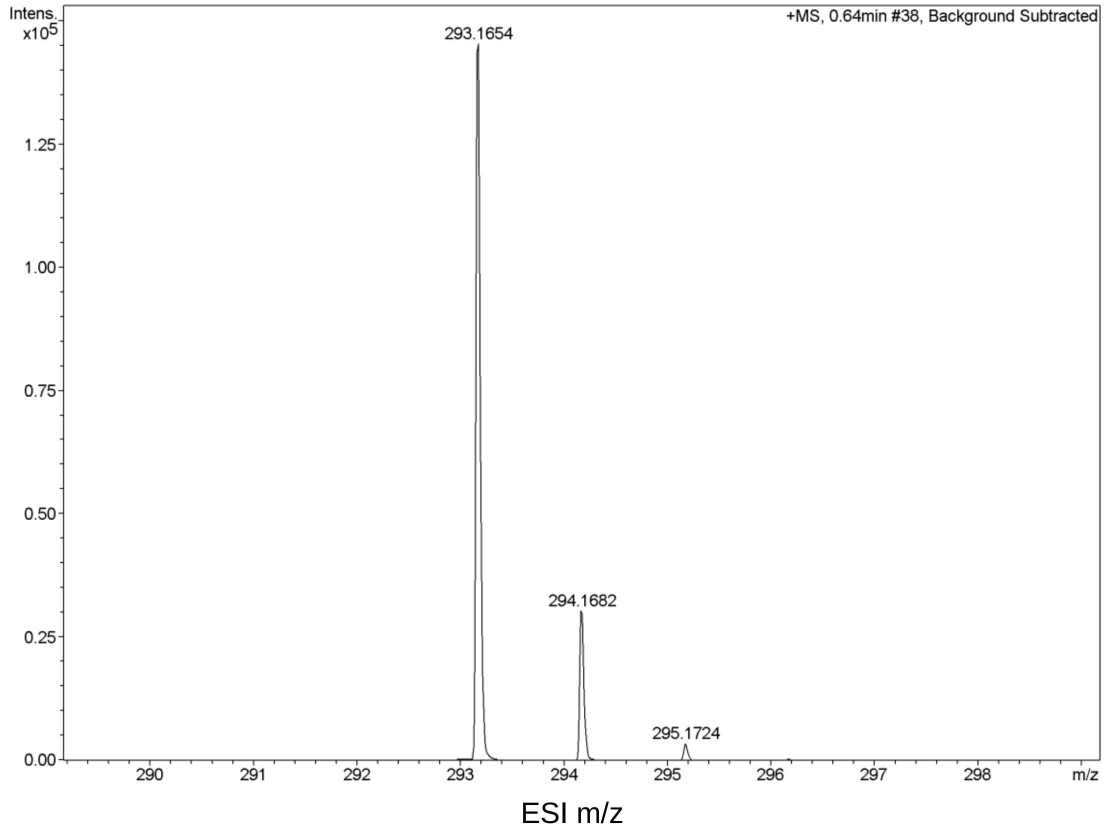


**Figure S20**. The HRMS of **3**.


[S1] Rafael Gómez-Bombarelli, Jennifer N Wei, David Duvenaud, José Miguel Hernández-Lobato, Benjamín Sánchez-Lengeling, Dennis Sheberla, Jorge Aguilera-Iparraguirre, Timothy D Hirzel, Ryan P Adams, and Alán Aspuru-Guzik. Automatic chemical design using a datadriven continuous representation of molecules. ACS central science, 4(2):268–276, 2018.

[S2] Mario Krenn, Florian Häse, AkshatKumar Nigam, Pascal Friederich, and Alan Aspuru-Guzik. Self-referencing embedded strings (selfies): A 100% robust molecular string representation. Machine Learning: Science and Technology, 1(4):045024, 2020.

[S3] A Nigam, Robert Pollice, Mario Krenn, Gabriel dos Passos Gomes, and Alan Aspuru-Guzik. Beyond generative models: Superfast traversal, optimization, novelty, exploration and discovery (stoned) algorithm for molecules using selfies. 2020.

[S4] Wengong Jin, Regina Barzilay, and Tommi Jaakkola. Junction tree variational autoencoder for molecular graph generation. In International Conference on Machine Learning, pages 2323–2332. PMLR, 2018.

[S5] Wengong Jin, Regina Barzilay, and Tommi Jaakkola. Hierarchical generation of molecular graphs using structural motifs. In International Conference on Machine Learning, pages 4839–4848. PMLR, 2020.

[S6] Junyan Jiang, Gus G Xia, Dave B Carlton, Chris N Anderson, and Ryan H Miyakawa. Transformer vae: A hierarchical model for structure-aware and interpretable music representation learning. In ICASSP 2020-2020 IEEE International Conference on Acoustics, Speech and Signal Processing (ICASSP), pages 516–520. IEEE, 2020.

[S7] Danyang Liu and Gongshen Liu. A transformer-based variational autoencoder for sentence generation. In 2019 International Joint Conference on Neural Networks (IJCNN), pages 1–7. IEEE, 2019.

[S8] https://github.com/hips/molecule-autoencoder.
